# Increased CHCHD2 expression promotes liver fibrosis in nonalcoholic steatohepatitis via Notch/osteopontin signaling

**DOI:** 10.1172/jci.insight.162402

**Published:** 2022-12-08

**Authors:** Yue Li, Wenjing Xiu, Jingwen Xu, Xiangmei Chen, Guangyan Wang, Jinjie Duan, Lei Sun, Ben Liu, Wen Xie, Guangyin Pu, Qi Wang, Chunjiong Wang

**Affiliations:** 1 Department of Pathology, Beijing Ditan Hospital, Capital Medical University, Beijing, China.; 2 Department of Physiology and Pathophysiology, The Province and Ministry Co-sponsored Collaborative Innovation Center for Medical Epigenetics, Tianjin Key Laboratory of Medical Epigenetics, NHC Key Laboratory of Hormones and Development, Chu Hsien-I Memorial Hospital and Tianjin Institute of Endocrinology, Tianjin Medical University, Tianjin, China.; 3 Center of Liver Diseases, Beijing Ditan Hospital, Capital Medical University, Beijing, China.

**Keywords:** Gastroenterology, Hepatology, Fibrosis, Hepatitis

## Abstract

Nonalcoholic steatohepatitis (NASH) is closely related to liver fibrosis. The role of coiled-coil-helix-coiled-coil-helix domain-containing 2 (CHCHD2) in NASH remains unknown. CHCHD2’s functions as a transcription factor have received much less attention than those in mitochondria. Herein, we systematically characterized the role of CHCHD2 as a transcription factor by chromatin immunoprecipitation sequencing and found its target genes were enriched in nonalcoholic fatty liver disease (NAFLD). Overall, CHCHD2 expression was found to be increased in the livers of patients with NAFLD and those of NASH mice. In line with these findings, CHCHD2 deficiency ameliorated NASH- and thioacetamide-induced liver fibrosis, whereas hepatocyte-specific CHCHD2 overexpression promoted liver fibrosis in NASH mice via Notch signaling. Specifically, CHCHD2-overexpressing hepatocytes activated hepatic stellate cells by upregulating osteopontin levels, a downstream mediator of Notch signals. Moreover, Notch inhibition attenuated CHCHD2 overexpression–induced liver fibrosis in vivo and in vitro. Then we found lipopolysaccharide-induced CHCHD2 expression in hepatocytes was reverted by verteporfin, an inhibitor that disrupts the interaction between Yes-associated protein (YAP) and transcriptional enhanced associate domains (TEADs). In addition, CHCHD2 levels were positively correlated with those of TEAD1 in human samples. In conclusion, CHCHD2 is upregulated via YAP/TAZ-TEAD in NASH livers and consequently promotes liver fibrosis by activating the Notch pathway and enhancing osteopontin production.

## Graphical abstract

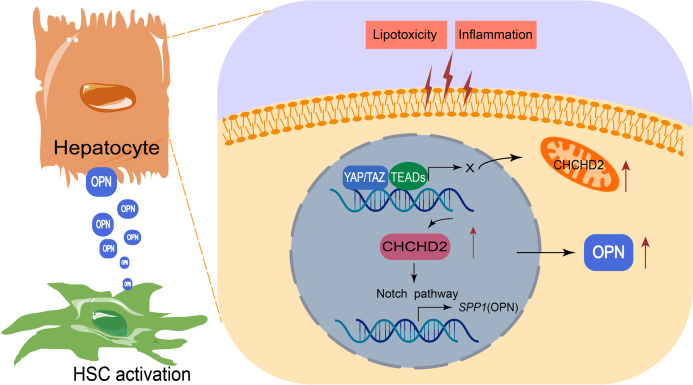

## Introduction

Nonalcoholic fatty liver disease (NAFLD) is classified into nonalcoholic simple fatty liver, nonalcoholic steatohepatitis (NASH), and NASH-related liver fibrosis according to pathological stage. NAFLD is one of the most important public health issues worldwide (1). Currently, the prevalence of NAFLD in the Asian population is around 25%, similar to that in many Western countries (2). Compared with simple steatosis, NASH is more likely to lead to liver fibrosis (1). However, effective approaches to prevent and treat NASH are still lacking. Therefore, we need to explore new targets for NASH treatment.

Coiled-coil-helix-coiled-coil-helix domain-containing 2 (CHCHD2) is a bi-organellar protein found in both the mitochondria and nucleus (3) and is also known as mitochondrial nuclear retrograde regulator 1. CHCHD2 was first identified by our research group in 2004 and submitted to the NCBI GenBank database as the hepatitis C virus non-structural protein 2 trans-regulated protein (https://www.ncbi.nlm.nih.gov/nuccore/AY605046).

The CHCHD2 protein contains an N-terminal mitochondria-targeting sequence and a twin Cx9C motif in the C-terminal CHCH domain. As a mitochondrial protein, CHCHD2 has been identified as a negative regulator of the mitochondrial apoptotic pathway, which acts by binding to the antiapoptotic protein Bcl-xL and inhibiting the accumulation of the proapoptotic protein Bax (4). In the nucleus, CHCHD2 regulates gene expression, including that of cytochrome c oxidase subunit 4-2 and itself as a transcription factor (5). Compared with its function as a mitochondrial protein, its function as a transcription factor is poorly studied. Our previous study found that CHCHD2 is highly expressed in hepatocellular carcinoma specimens (6). It has also been reported that NASH increases the risk of primary liver cancer (7). However, the effects of CHCHD2 on NASH remain unknown.

The fibrogenic crosstalk between hepatocytes and hepatic stellate cells (HSCs) is a major pathophysiological process in liver fibrosis during NAFLD progression (8). Activation of the Notch and Yes-associated protein (YAP)/transcriptional coactivator with PDZ-binding motif (TAZ)-transcriptional enhanced associate domain (TEAD) (the core components of the Hippo pathway) pathway in hepatocytes mediates HSC activation and promotes liver fibrosis (8). In hepatocytes, Notch activation promotes liver fibrosis by increasing osteopontin (OPN) production (9, 10). The TEAD family members (TEAD1–4) are key transcription factors of the Hippo pathway (11, 12). When the Hippo pathway is inhibited, YAP/TAZ enters the nucleus and binds with TEADs or other transcription factors to regulate gene expression (11). Recent studies have shown that the Hippo pathway is essential for NAFLD progression (13–15). However, it remains unknown whether there is an association between the Hippo and/or Notch pathways and CHCHD2.

In this study, we systematically characterized the role of CHCHD2 as a transcription factor by chromatin immunoprecipitation (ChIP) sequencing, which indicates it participates in NAFLD as a transcription factor. Notably, we investigated its effects on NAFLD and underlying molecular mechanisms in vitro and in vivo.

## Results

### CHCHD2 participates in NAFLD as a transcription factor.

**Figure 1:**
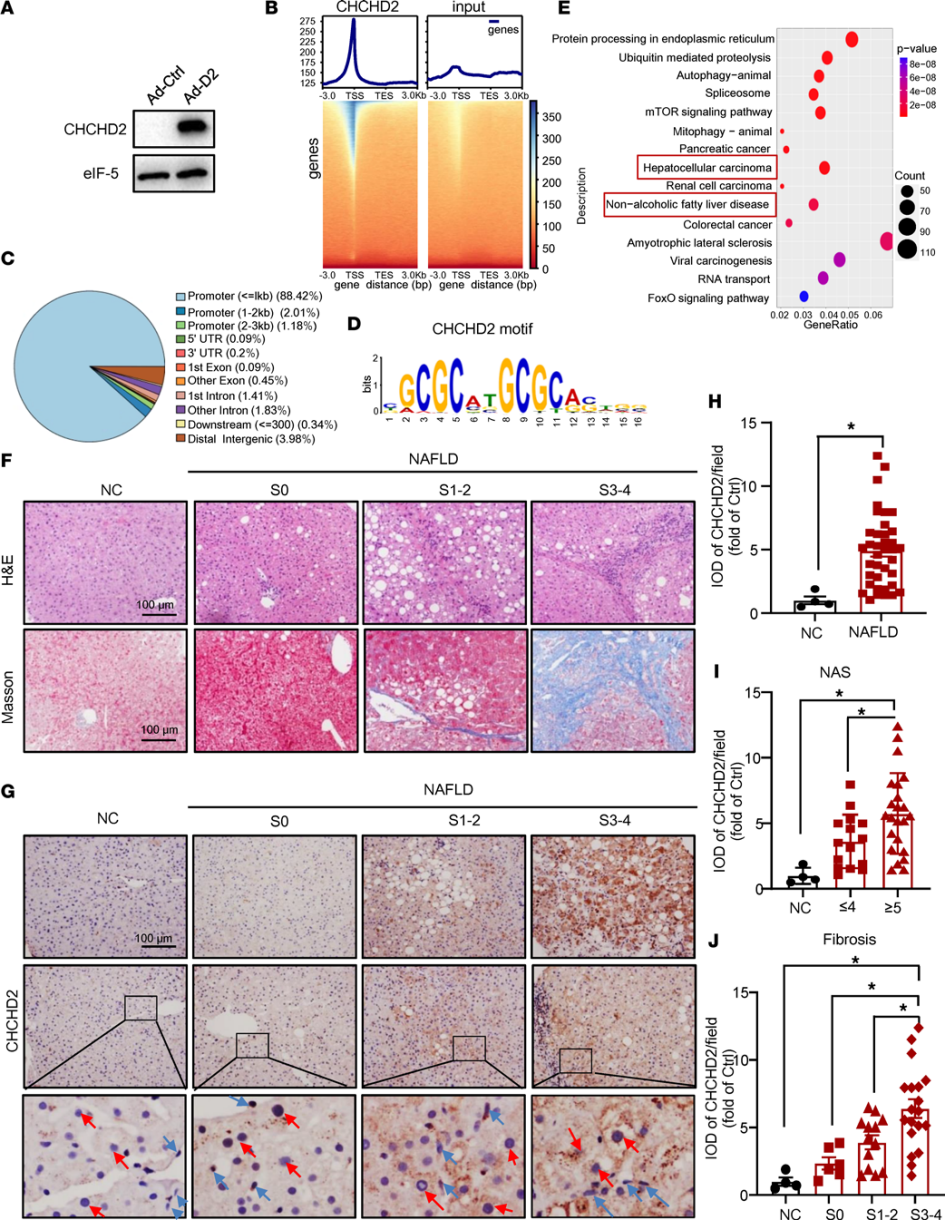
ChIP-sequencing analysis of CHCHD2 and the CHCHD2 expression in patients with NAFLD. (**A**–**E**) CHCHD2 was overexpressed in primary mouse hepatocytes by adenovirus. (**A**) Western blot analysis of protein level of CHCHD2; ChIP sequencing was performed by anti-CHCHD2 antibody in CHCHD2-overexpressed primary mouse hepatocytes. (**B**) Density plot and heatmap of CHCHD2 peak locations in relation to the transcriptional start site (TSS), (**C**) peak distribution, and (**D**) the CHCHD2 motif were analyzed. TES, transcription end site. (**E**) KEGG enrichment of annotated peaks and top 15 enriched KEGG pathways based on *P* value. (**F**) H&E and Masson’s trichome staining of liver sections of healthy controls and patients with NAFLD at different stages of fibrosis. (**G**) Representative immunohistochemical staining of CHCHD2 of liver sections of healthy controls (*n* = 4) and NAFLD patients (*n* = 38) at different stages of fibrosis. Red arrow, representative hepatocyte; blue arrow, representative nonparenchymal cell. Inset original magnification, ×25. (**H**–**J**) Quantification of integrated optical density of CHCHD2. (**H**) Integrated optical density (IOD) of CHCHD2 of liver sections from patients with NAFLD and healthy controls. (**I**) IOD of CHCHD2 of liver sections from healthy controls (*n* = 4) and NAFLD patients with NAS score ≤ 4 (*n* = 15) or ≥ 5 (*n* = 23). (**J**) IOD of CHCHD2 of liver sections from healthy controls (*n* = 4) and NAFLD patients with S0 (*n* = 6), S1–2 (*n* = 13), and S3–4 (*n* = 19) fibrosis. *P < 0.05. NC, normal control. (**H**) *P* value was from unpaired *t* test; (**I** and **J**) *P* values were from a 1-way ANOVA with a post hoc Fisher’s least significant difference (LSD) test.

Compared with its function as a mitochondrial protein, CHCHD2’s role as a transcription factor is poorly studied. Thus, herein, we forced CHCHD2 overexpression in primary mouse hepatocytes (Figure 1A) and performed ChIP sequencing. Most CHCHD2 peaks were located close to transcriptional start sites (Figure 1B), with more than 90% of these peaks being located in promoter regions (Figure 1C). The CHCHD2 binding motif was analyzed based on the peaks (Figure 1D). The top 15 enriched Kyoto Encyclopedia of Genes and Genomes (KEGG) pathways (based on the *P* value) showed that CHCHD2 target genes were involved in 2 liver diseases: NAFLD and hepatocellular carcinoma (Figure 1E). Consistently, we previously reported that CHCHD2 is highly expressed in hepatocellular carcinoma (6); however, to date, the role of CHCHD2 in NAFLD remains unknown.

### Expression of CHCHD2 is increased in patients with NAFLD and is positively related to fibrosis.

To explore CHCHD2’s role in NAFLD, liver tissue samples were collected from 38 patients with NAFLD and 4 patients with normal livers and CHCHD2 expression was evaluated by immunohistochemical staining. According to the NASH Clinical Research Network (NASH-CRN) system, liver samples were divided into 4 groups: control, NAFLD fibrosis stage 0 (S0), NAFLD fibrosis stages 1–2 (S1–2), and NAFLD fibrosis stages 3–4 (S3–4). The general characteristics of these patients are summarized in Table 1. Liver samples from patients with NAFLD with fibrosis showed increased collagen deposition, ballooning degeneration, and inflammatory cell infiltration, as indicated by H&E and Masson’s trichome staining (Figure 1F). We found CHCHD2 levels were increased in patients with NAFLD compared with that in controls (Figure 1, G and H). Of note, CHCHD2 expression was found to be higher in patients with NAFLD with NAS score ≥ 5 than in those with NAS score ≤ 4 (Figure 1I), which suggested that CHCHD2 was related to NASH (16). Moreover, CHCHD2 expression was associated with the stage of fibrosis in these patients; patients with S3–4 fibrosis showed higher CHCHD2 expression than the other groups (Figure 1, G and J). The immunohistochemical staining of CHCHD2 also showed CHCHD2 was upregulated in both hepatocytes and some nonparenchymal cells in NASH liver (Figure 1G). These results suggest that CHCHD2 expression may be involved in NAFLD development and fibrosis occurrence in patients with NAFLD.

### CHCHD2 is highly expressed in NASH mouse liver.

**Figure 2:**
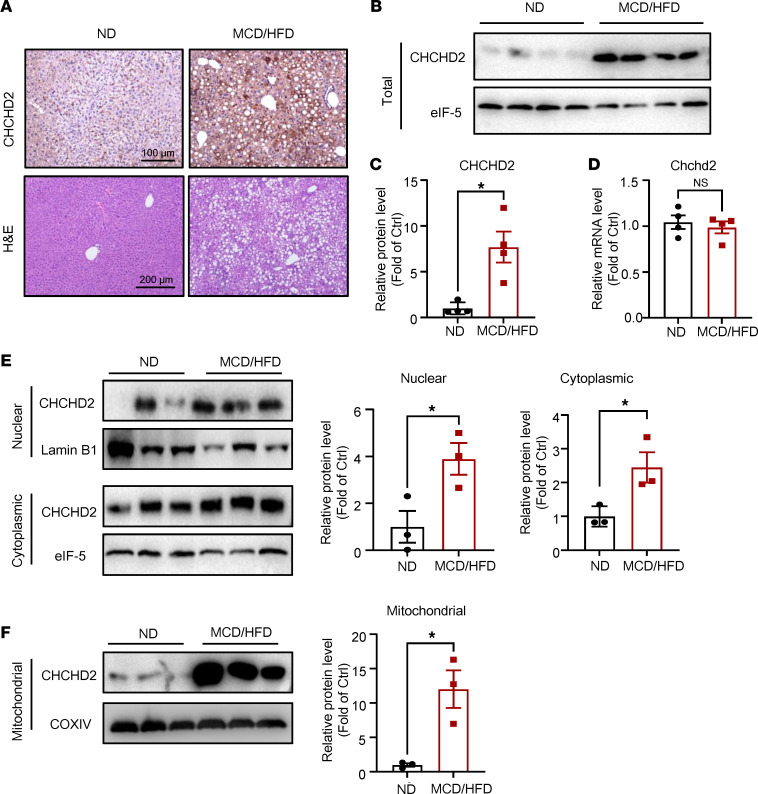
CHCHD2 is upregulated in NASH mouse liver. Wild-type mice were fed a normal diet (ND) or a methionine/choline-deficient plus high-fat diet (MCD/HFD) for 4 weeks. (**A**) Representative immunohistochemical staining of CHCHD2 and H&E staining of the mouse liver sections. (**B** and **C**) Western blot analysis of protein level of CHCHD2 in liver. (**D**) Quantitative PCR (qPCR) analysis of mRNA level of *Chchd2* in the liver. (**A**–**D**) *n* = 4 per group. (**E**) Analysis of protein levels of CHCHD2 in nuclear and cytoplasmic fractions by Western blot. (**F**) Western blot analysis of CHCHD2 levels in mitochondria of liver tissue. (**E** and **F**) *n* = 3 per group. *P < 0.05. *P* values were from unpaired *t* test.

We further found that the protein level of CHCHD2 was greatly increased in livers of mice with NASH induced by methionine/choline-deficient plus 45% fat diet (MCD/HFD) feeding for 4 weeks, as evidenced by both immunohistochemical staining and Western blotting (Figure 2, A–C). However, the mRNA expression levels were comparable between ND- and MCD/HFD-fed mice (Figure 2D). Moreover, hepatic CHCHD2 protein was also upregulated in mice with NASH induced by 24-week feeding with a high-fructose, high-palmitate, and high-cholesterol (FPC) diet (Supplemental Figure 1A; supplemental material available online with this article; https://doi.org/10.1172/jci.insight.162402DS1) (15, 17). CHCHD2 is expressed in both the mitochondria and nucleus; thus, we next explored the subcellular distribution of increased CHCHD2 in the liver. First, we isolated nuclear and cytoplasmic proteins and found that CHCHD2 was upregulated in both fractions (Figure 2E). We found that CHCHD2 was also increased in the mitochondrial protein (Figure 2F). The high purity of mitochondrial and nuclear proteins was evidenced by protein levels of COXIV and Lamin B1 (Supplemental Figure 1B). The immunofluorescence staining of CHCHD2 further supported that its protein level in hepatocytes of NASH mouse liver was increased inside and outside the nuclei (Supplemental Figure 1C). These data indicate that CHCHD2 is significantly upregulated at the total protein level in the livers of mice with NASH.

### CHCHD2 deletion attenuates liver fibrosis in NASH and thioacetamide-treated mice.

**Figure 3:**
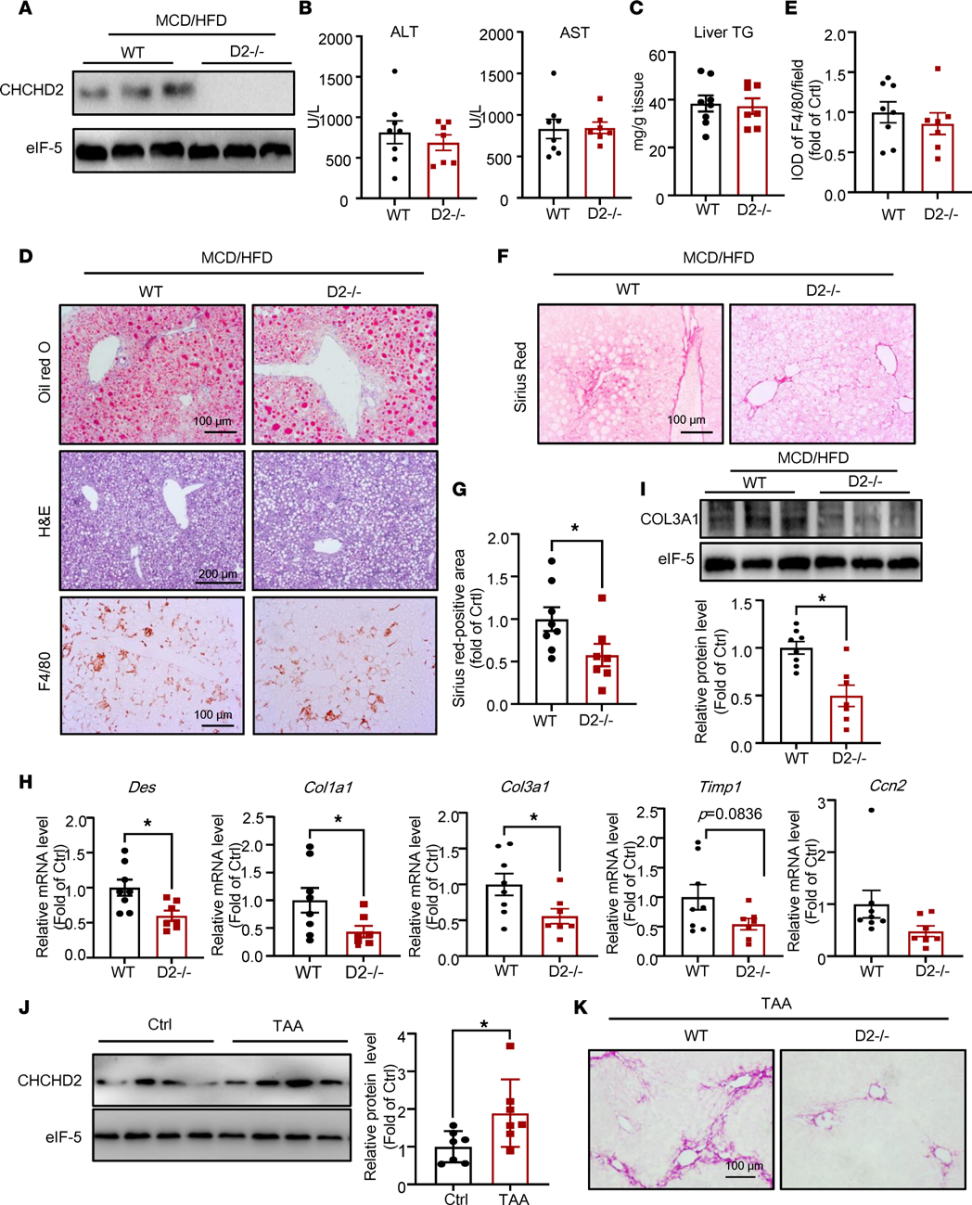
CHCHD2 knockout improves fibrosis in NASH and TAA-treated mouse livers. (**A**–**I**) CHCHD2-knockout mice and the littermate control were fed with an MCD/HFD for 4 weeks. (**A**) Western blot analysis of protein level of CHCHD2 in liver. (**B**) Plasma ALT and AST level. (**C**) Liver tissue TG content. Oil Red O, H&E, and immunohistochemical staining of F4/80 in mouse livers (**D**) and integrated optical density (IOD) of F4/80 (**E**). Sirius red staining of liver sections (**F**) and positive area of Sirius red staining (**G**). (**H**) Quantitative PCR (qPCR) analysis of mRNA levels of *Des*, *Col1a1*, *Col3a1*, *Timp1*, and *Ccn2* in liver. (**I**) Western blot analysis of COL3A1 level. *n* = 7–8 per group. (**J**) Western blot analysis of CHCHD2 in liver of wild-type mice treated with TAA for 7 weeks (*n* = 7 per group). (**K**) Sirius red staining of liver sections of CHCHD2-knockout mice and the littermate control treated with TAA (*n* = 6–7 per group). *P < 0.05. *P* values were from unpaired *t* test. D2-/-, CHCHD2 knockout.

To demonstrate the effects of CHCHD2 on NASH, we developed CHCHD2-knockout mice (Supplemental Figure 2A). The morphology of livers from CHCHD2-knockout mice did not differ from that of wild-type mice when examined by H&E staining (Supplemental Figure 2B). The body weight, weight of liver, inguinal white adipose tissue (iWAT), and epididymal white adipose tissue (eWAT) were comparable between the knockout mice and wild-type mice on a normal diet (Supplemental Figure 2, C and D). We then fed CHCHD2-knockout mice and the litter mate controls with MCD/HFD diet for 4 weeks (Figure 3A). The body weight, liver weight, and plasma ALT and AST levels were not changed by CHCHD2 deletion in the NASH mouse model (Supplemental Figure 3A and Figure 3B). Previous studies have suggested an antiapoptotic role of CHCHD2; however, we found that CHCHD2 deletion did not increase cell apoptosis in NASH mouse liver, as evidenced by the TUNEL staining and the unchanged level of cleaved caspase-3 (Supplemental Figure 3, B and C). Hepatic triglyceride content was not changed by CHCHD2 deletion (Figure 3C). Moreover, Oil Red O staining and immunohistochemical staining of F4/80 showed that CHCHD2 knockout did not affect steatosis and macrophage infiltration in the livers of mice with NASH (Figure 3, D and E). However, Sirius red staining revealed that liver fibrosis was decreased by CHCHD2 deletion (Figure 3, F and G), which was further evidenced by the lower expression of fibrotic markers, including desmin (*Des*), collagen type I α1 (*Col1a1*), collagen type III α1 (*Col3a1*), and tissue inhibitor of metalloproteinase 1 (*Timp1*) (*P* = 0.0836) (Figure 3H). Consistently, the protein level of COL3A1 was also decreased in CHCHD2-knockout mice (Figure 3I). These data indicate that CHCHD2 deficiency may improve liver fibrosis in NASH mice.

Additionally, we found that CHCHD2 was also upregulated in thioacetamide-induced (TAA-induced) fibrotic livers (Figure 3J). CHCHD2 knockout greatly suppressed TAA-induced liver fibrosis, as evidenced by Sirius red staining (Figure 3K) and reduced expression levels of *Des*, *Timp1*, transforming growth factor-β (*Tgfb*), *Col1a1* (*P* = 0.055), and *Col3a1* (*P* = 0.054) (Supplemental Figure 4).

### Elevated hepatocyte CHCHD2 accelerates the evolution of hepatic fibrosis.

**Figure 4:**
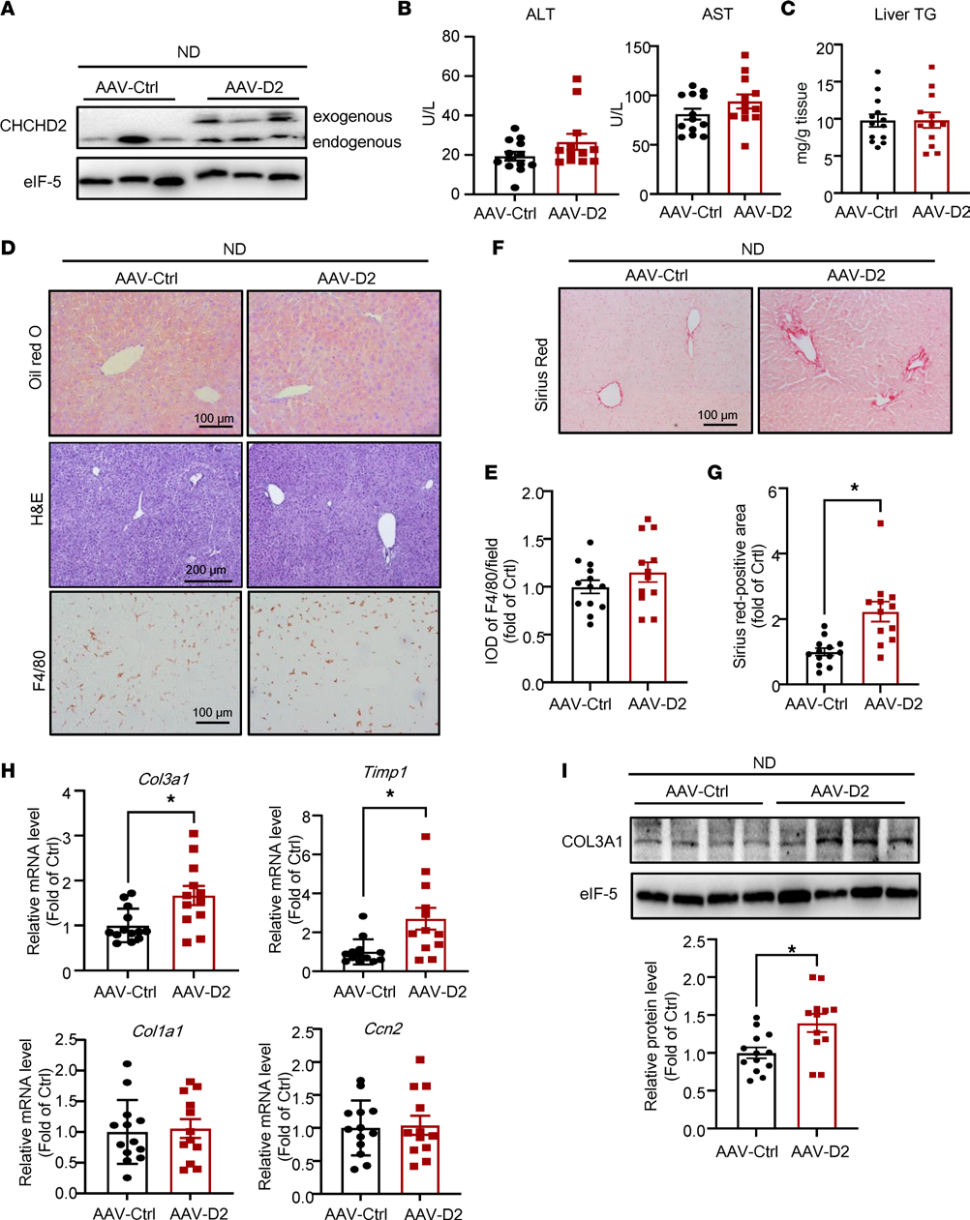
Hepatocyte-specific overexpression of CHCHD2 increases liver collagen deposition under ND. Mice were injected with AAV-CHCHD2-flag bearing *TBG* promoter or control AAV for 38 days. (**A**) Western blot analysis of protein levels of CHCHD2 in liver. (**B**) Plasma ALT and AST levels. (**C**) Liver TG content. (**D** and **E**) Oil Red O, H&E, and immunohistochemical staining of F4/80 in mouse livers (**D**) and integrated optical density (IOD) of F4/80 (**E**). (**F** and **G**) Sirius red staining of liver sections (**F**) and positive area of Sirius red staining (**G**). (**H**) Quantitative PCR (qPCR) analysis of mRNA levels of *Col3a1*, *Timp1*, *Col1a1*, and *Ccn2*. (**I**) Western blot analysis of COL3A1 level. *n* = 12–13 per group. *P < 0.05. *P* values were from unpaired *t* test. AAV-D2, AAV-CHCHD2.

CHCHD2 is expressed in various cell types in the liver. As shown in liver sections of patients and mice, hepatocyte CHCHD2 was upregulated in this study (Figure 1G and Figure 2A). We next investigated the effects of hepatocyte-specific overexpression of CHCHD2 on NASH. Mice were injected with adeno-associated virus– (AAV-) CHCHD2-flag with the thyroxine binding globulin (*TBG*) promoter to overexpress CHCHD2 specifically in hepatocytes, and control mice were injected with control AAV. The flag was detected only in the livers of mice injected with AAV-CHCHD2 and not in the eWAT, iWAT, and pancreas (Supplemental Figure 5A). Then, 10 days after AAV injection, the mice were fed an ND or MCD/HFD for 4 weeks. The body and liver weight were not changed by CHCHD2 overexpression (Supplemental Figure 5B). Liver CHCHD2 expression in mice on ND was analyzed by Western blotting (Figure 4A). In these mice, CHCHD2 overexpression did not affect plasma ALT and AST levels (Figure 4B). Hepatic TG content determination and Oil Red O staining showed that hepatic lipid content remained unchanged in CHCHD2-overexpressed mice (Figure 4, C and D). Macrophage infiltration also did not differ as evidenced by the immunohistochemical staining of F4/80 (Figure 4, D and E). Of note, Sirius red staining demonstrated increased collagen deposition after CHCHD2 overexpression (Figure 4, F and G). In addition, CHCHD2 overexpression increased the expression of the fibrotic markers *Col3a1* and *Timp1* in ND-fed mice (Figure 4H). The protein levels of COL3A1 were also upregulated in the livers of CHCHD2-overexpressing mice (Figure 4I).

**Figure 5:**
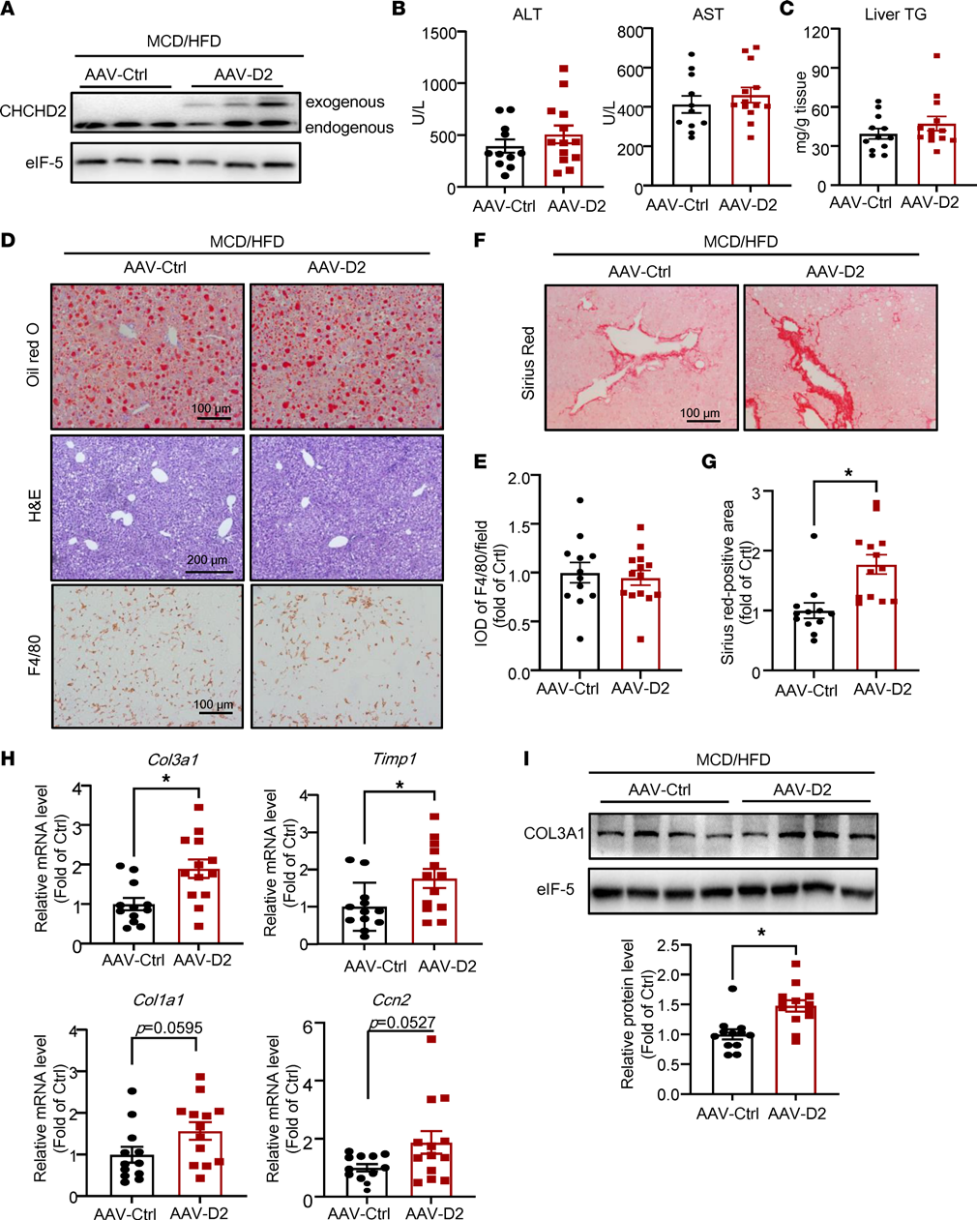
Hepatocyte-specific overexpression of CHCHD2 promotes liver fibrosis in NASH mice. Mice were injected with AAV-CHCHD2-flag bearing *TBG* promoter or control AAV for 10 days and then fed with MCD/HFD for 4 weeks. (**A**) Western blot analysis of protein levels of CHCHD2 in the liver. (**B**) Plasma ALT and AST levels. (**C**) Liver TG content. (**D** and **E**) Oil Red O, H&E, and immunohistochemical staining of F4/80 in mouse livers (**D**) and integrated optical density (IOD) of F4/80 (**E**). (**F** and **G**) Sirius red staining of liver sections (**F**) and positive area of Sirius red staining (**G**). (**H**) Quantitative PCR (qPCR) analysis of mRNA levels of *Col3a1*, *Timp1*, *Col1a1*, and *Ccn2*. (**I**) Western blot analysis of COL3A1 level. *n* = 12–13 per group (for ALT and AST, 1 sample in AAV-control (AAV-Ctrl) was missing because of insufficient volume of plasma). *P < 0.05. *P* values were from unpaired *t* test. AAV-D2, AAV-CHCHD2.

In MCD/HFD-fed mice, CHCHD2 overexpression did not affect plasma ALT and AST levels (Figure 5, A and B), lipid accumulation (Figure 5, C and D), or macrophage infiltration (Figure 5, D and E). Notably, hepatocyte-specific CHCHD2 overexpression further exacerbated liver fibrosis in NASH mice, as shown by Sirius red staining (Figure 5, F and G). In support of these observations, the hepatic mRNA levels of *Timp1*, *Col3a1*, *Col1a1* (*P* = 0.0595), and connective tissue growth factor (*Ccn2*; *P* = 0.0527) and the protein level of COL3A1 were higher in CHCHD2-overexpressed mice than in control mice (Figure 5, H and I).

### CHCHD2-overexpressing hepatocytes promote HSC activation via the Notch/OPN pathway.

**Figure 6:**
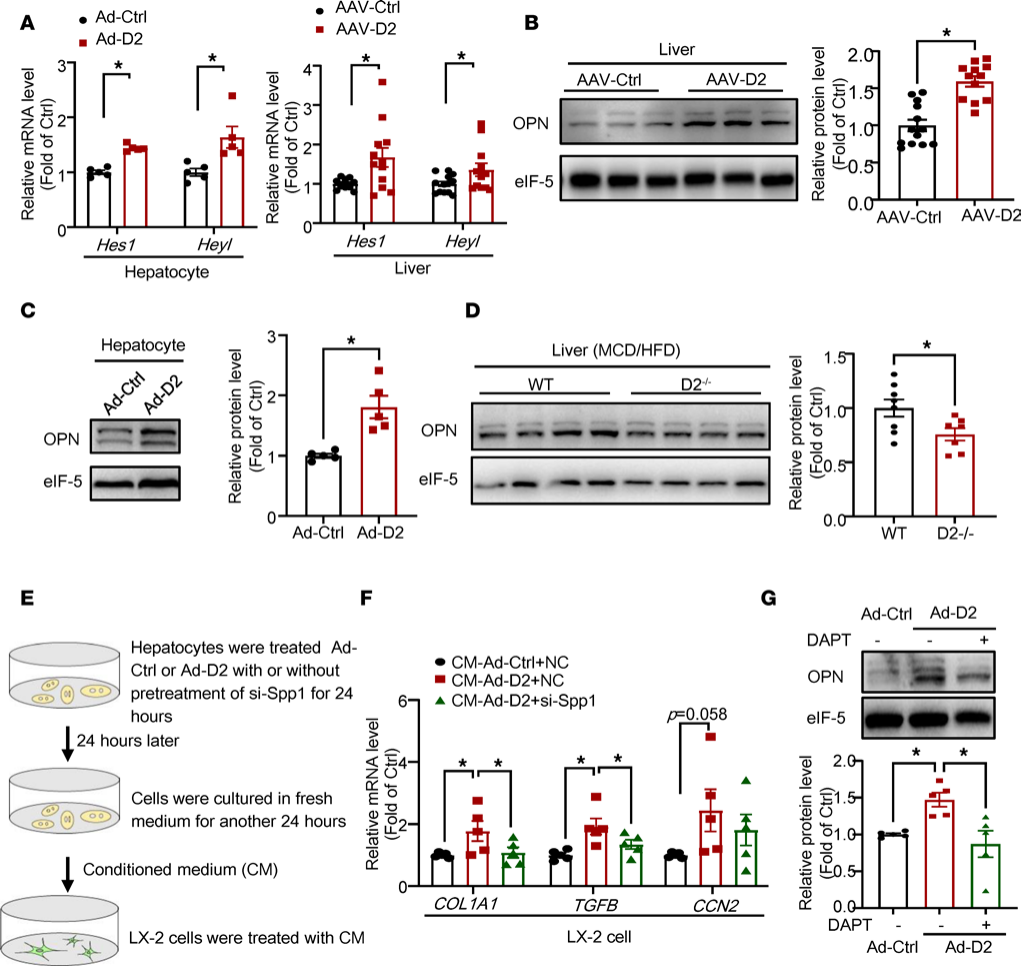
The effects of CHCHD2-overexpressed hepatocytes on HSC activation. (**A**) Quantitative PCR (qPCR) analysis of mRNA level of *Hes1* and *Heyl* in CHCHD2-expressing adenovirus–treated (Ad-CHCHD2–treated) hepatocytes (*n* = 5 independent experiments) and AAV-CHCHD2–treated mouse liver (*n* = 12–13 per group). (**B**) Western blot analysis of protein level of OPN in AAV-CHCHD2–treated mouse liver (*n* = 12–13 per group). (**C**) Western blot analysis of protein level of OPN in Ad-CHCHD2–treated hepatocytes (*n* = 5 independent experiments). (**D**) Western blot analysis of protein level of OPN in CHCHD2-knockout mouse liver (*n* = 7–8 per group). (**E** and **F**) LX-2 cells were treated with conditioned medium from primary mouse hepatocytes treated with Ad-Ctrl or Ad-CHCHD2 with or without pretreatment of Spp1 siRNA (si-*Spp1*): qPCR analysis of mRNA levels of *COL1A1*, *TGFB*, and *CCN2*. (**G**) Primary mouse hepatocytes treated with Ad-Ctrl or Ad-CHCHD2 with or without pretreatment of DAPT (5 μM), then Western blot analysis of the protein level of OPN. (**E**–**G**) *n* = 5 independent experiments. *P < 0.05. CM, conditioned medium; NC, negative control; Ad-D2, Ad-CHCHD2. (**A**–**D**) *P* values were from unpaired *t* test; (**F** and **G**) *P* values were from a 1-way ANOVA with a post hoc Fisher’s least significant difference (LSD) test.

Next, we investigated the mechanisms underlying CHCHD2-induced liver fibrosis. ChIP-sequencing analysis (related to Figure 1, A–E) showed that CHCHD2 binds to promoters of Notch pathway genes, including *Notch2*, *Notch4*, presenilin 1 (*Psen1*), presenilin enhancer γ secretase subunit (*Psenen*), aph1 homolog C γ secretase subunit (*Aph1c*), hes family bHLH transcription factor 1 (*Hes1*), recombination signal binding protein for immunoglobulin κ J region (*Rbpj*), CREB binding protein (*Crebbp*), dishevelled segment polarity protein 1 (*Dvl1*), NUMB endocytic adaptor protein (*Numb*), C-terminal binding protein 1 (*Ctbp1*), histone deacetylase 1 (*Hdac1*), ataxin 1 (*Atxn1*), and lysine acetyltransferase 2B (*Kat2b*) (Supplemental Table 1). Among these genes, we found that the expression of *Psen1*, *Psenen*, *Rbpj*, *Dvl1*, and *Ctbp1* was increased by CHCHD2 overexpression with unchanged *Notch2*, *Notch4*, and *Crebbp* levels (Supplemental Figure 6). As *Psen1*, *Psenen*, and *Rbpj* positively regulate Notch signaling whereas *Dvl1* and *Ctbp1* negatively regulate Notch signaling, we analyzed the mRNA levels of *Hes1* and hes related family bHLH transcription factor with YRPW motif like (*Heyl*), the target genes of the Notch pathway, to show the overall effects of CHCHD2 on Notch signaling. The increased expression levels of *Hes1* and *Heyl* suggest that the Notch pathway is activated by CHCHD2 (Figure 6A).

It is known that Notch signals in hepatocytes activate HSCs via paracrine induction of OPN secretion, which subsequently promotes liver fibrosis in NASH (10, 18, 19). Noteworthily, CHCHD2 overexpression was found to induce OPN (encoded by gene secreted phosphoprotein 1, *Spp1*) expression in hepatocytes and mouse liver (Figure 6, B and C), whereas CHCHD2 knockdown decreased the OPN levels in NASH mouse liver (Figure 6D). In addition, conditioned medium from CHCHD2 overexpression hepatocytes promoted HSC activation, which was repressed by *Spp1* knockdown (Supplemental Figure 7 and Figure 6, E and F). We further found that the Notch inhibitor DAPT suppressed CHCHD2-induced OPN expression (Figure 6G). Taken together, these data indicate that CHCHD2 increases OPN expression via Notch activation and promotes liver fibrosis.

### The inhibition of Notch signaling attenuates liver fibrosis exacerbated by CHCHD2 overexpression in NASH mice.

**Figure 7:**
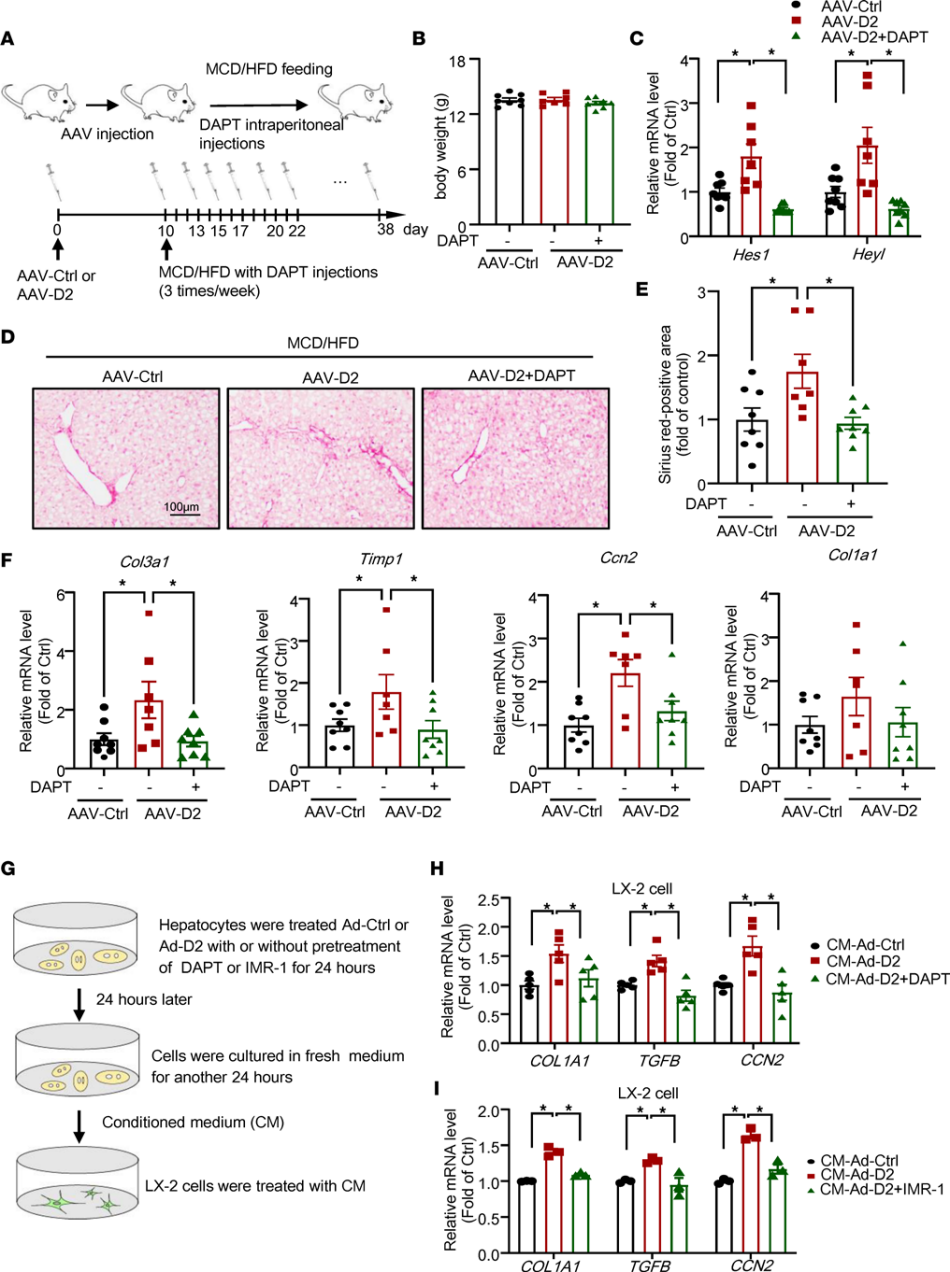
Effects of Notch inhibition on CHCHD2 overexpression–promoted liver fibrosis in NASH mice. Mice were injected with AAV-CHCHD2-flag bearing a *TBG* promoter or control AAV for 10 days and then fed with an MCD/HFD for 4 weeks; during the NASH diet feeding period, mice were intraperitoneally injected with the Notch inhibitor DAPT (10 mg/kg) or the control solvent 3 times per week (**A**). (**B**) Body weight. (**C**) Quantitative PCR (qPCR) analysis of *Hes1* and *Heyl* mRNA levels in livers. (**D**) Sirius red staining of liver sections and (**E**) positive area of Sirius red staining. (**F**) qPCR analysis of mRNA levels of *Col3a1*, *Timp1*, *Col1a1*, and *Ccn2*; *n* = 7–8 per group. (**G**–**I**) Primary mouse hepatocytes treated with Ad-Ctrl or Ad-CHCHD2 with or without pretreatment with DAPT (5 μM) or IMR-1 (5 μM); then, LX-2 cells were treated with conditioned medium from these hepatocytes, and the mRNA levels of *COL1A1*, *TGFB*, and *CCN2* were analyzed by qPCR. (**H**) *n* = 5 independent experiments; (**I**) *n* = 3 independent experiments. *P < 0.05. *P* values were from a 1-way ANOVA with a post hoc Fisher’s least significant difference (LSD) test.

To investigate the role of Notch signaling in CHCHD2 overexpression–induced liver fibrosis in NASH mice, we treated mice with the Notch inhibitor DAPT (Figure 7A). The body weight was not changed by DAPT treatment (Figure 7B). We found that the *Hes1* and *Heyl* level was increased by CHCHD2 overexpression, which was decreased by DAPT treatment (Figure 7C). Sirius red staining revealed that Notch inhibition substantially attenuated liver fibrosis exacerbated by CHCHD2 overexpression in MCD/HFD-fed mice (Figure 7, D and E). In line with this finding, the elevated expression of *Col3a1*, *Timp1*, and *Ccn2* in CHCHD2-overexpressing mice was also repressed by Notch inhibition (Figure 7F). Then, we investigated the effects of Notch signaling on crosstalk involving CHCHD2-overexpressing hepatocytes and HSCs by 2 Notch inhibitors, DAPT and IMR-1. LX-2 cells were treated with conditioned medium from CHCHD2-overexpressing hepatocytes with or without pretreatment with DAPT or IMR-1. We found Notch inhibition by DAPT or IMR-1 in hepatocytes attenuated CHCHD2-overexpressing hepatocyte–induced HSC activation (Figure 7, G–I).

### Palmitate and LPS induce elevated CHCHD2 expression related to increased TEAD1 in NASH liver.

**Figure 8:**
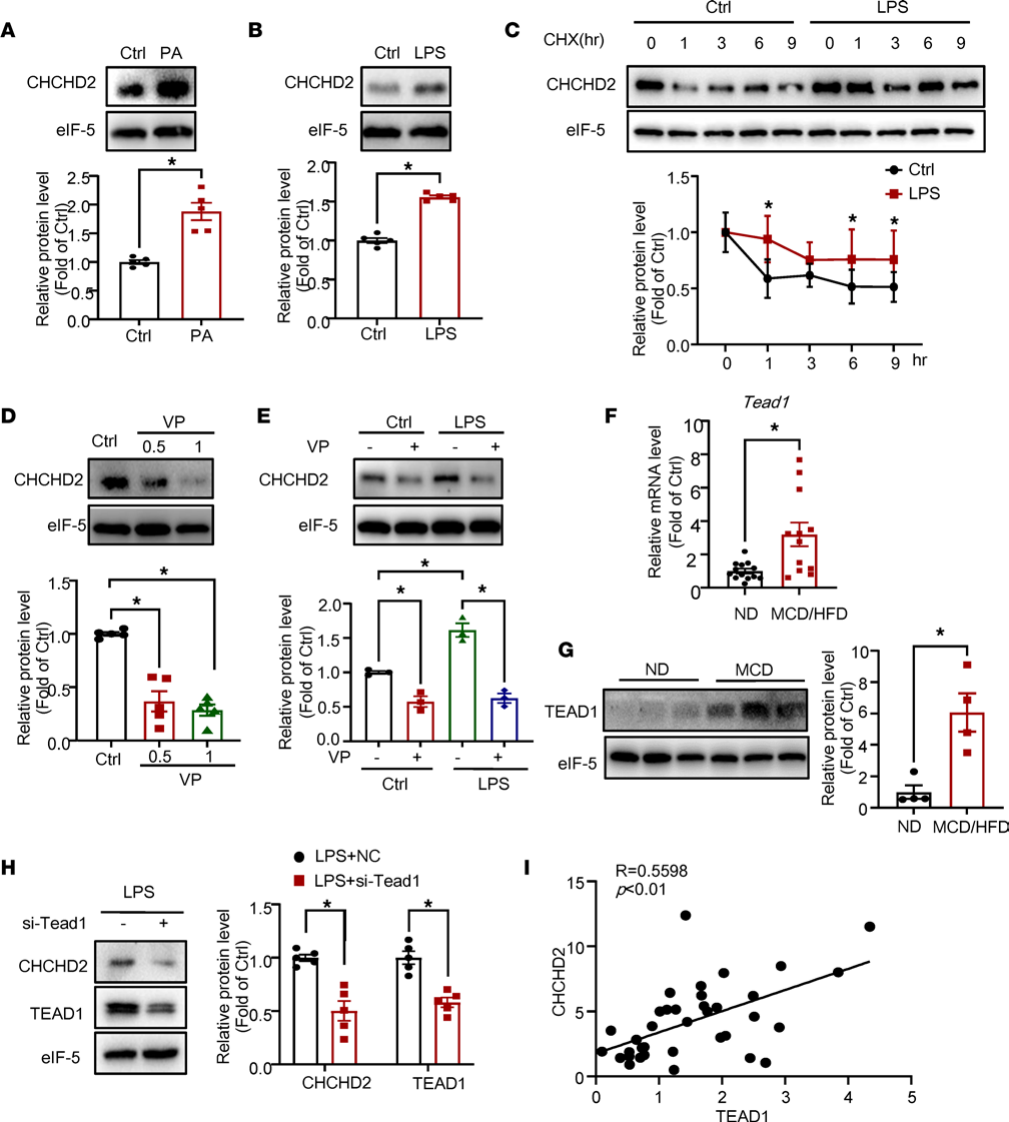
CHCHD2 expression is upregulated by palmitate and LPS and is suppressed by verteporfin. (**A**–**E**) Western blot analysis of protein level of CHCHD2: primary murine hepatocytes were treated with 100 μM palmitate for 48 hours (**A**) or 100 ng/mL LPS for 48 hours (*n* = 5 independent experiment) (**B**); (**C**) primary murine hepatocytes were treated with 100 ng/mL LPS and 50 ng/mL cycloheximide (CHX) for 0, 1, 3, 6, or 9 hours (*n* = 5 independent experiments); (**D**) primary murine hepatocytes were treated with 0.5 or 1 nM verteporfin for 48 hours (*n* = 5 independent experiments); (**E**) primary murine hepatocytes were treated with LPS for 48 hours with or without pretreatment with 1 nM verteporfin for 0.5 hours (*n* = 3 independent experiments). (**F**) Quantitative PCR (qPCR) analysis of mRNA levels of *Tead1* in MCD/HFD-fed mouse liver (*n* = 12–13 per group). (**G**) Western blot analysis of protein level of TEAD1 in MCD/HFD-fed mouse liver (*n* = 4 per group). (**H**) Hepatocytes were transfected with siRNA targeting *Tead1* and then treated with LPS. Western blot analysis of protein levels of CHCHD2 and TEAD1 (*n* = 5 independent experiments). (**I**) Correlation analysis of CHCHD2 and TEAD1 expression based on the immunohistochemical staining in patient livers (*n* = 33; compare to Figure 1B, 9 human liver samples were not stained with TEAD1 antibody because the liver sections ran out). *P < 0.05. (**A**, **B**, and **F**–**H**) *P* values were from unpaired *t* test; (**C**) *P* values were from a 2-way ANOVA with a post hoc Fisher’s least significant difference (LSD) test; (**D** and **E**) *P* values were from a 1-way ANOVA with a post hoc Fisher’s LSD test. VP, verteporfin; PA, palmitate.

To study the regulatory mechanisms for elevated CHCHD2 expression in hepatocytes of NASH liver, we treated primary hepatocytes with palmitate to induce lipotoxicity or LPS to induce inflammation. Palmitate and LPS treatment significantly increased the protein expression of CHCHD2 in hepatocytes (Figure 8, A and B). Consistent with the findings in the livers of mice with NASH, the mRNA level of *Chchd2* in hepatocytes was not changed by palmitate or LPS treatment (Supplemental Figure 8A). Then we treated hepatocyte with cycloheximide (CHX) to prevent the translation of proteins and found LPS increased the stability of CHCHD2 (Figure 8C).

The Hippo pathway has been demonstrated to play important roles in NASH and liver carcinogenesis (20). Both lipotoxicity and inflammation increase YAP/TAZ-TEAD activity in hepatocytes and promote liver fibrosis (14, 21). We investigated whether CHCHD2 expression could be regulated by the Hippo pathway. Verteporfin is an inhibitor that disrupts the interaction between YAP and TEADs and leads to decreased expression of TEAD target genes (22). We treated hepatocytes with verteporfin and found that it significantly downregulated CHCHD2 expression in hepatocytes (Figure 8D). In addition, LPS-induced CHCHD2 upregulation was also suppressed by verteporfin treatment (Figure 8E). The mRNA level of *Chchd2* was not changed by verteporfin treatment (Supplemental Figure 8B), which indicates that CHCHD2 was not directly upregulated by TEADs at the transcriptional level.

In addition, we found that the mRNA and protein levels of TEAD1 were significantly increased in livers of mice with NASH (Figure 8, F and G). However, *Tead3* was downregulated, and *Tead2* and *Tead4* were unchanged in livers of MCD/HFD-fed mice (Supplemental Figure 8C). Moreover, the LPS-induced CHCHD2 expression was decreased by *Tead1* knockdown (Figure 8H). Finally, immunohistochemical staining revealed that the expression level of CHCHD2 was positively correlated with nucleus TEAD1 in human liver samples (Figure 8I and Supplemental Figure 8D). These data indicate that elevated CHCHD2 expression may result from increased TEAD1 expression in NASH livers.

## Discussion

There has been an increase in the global prevalence of NAFLD. Our research shows that patients with NAFLD in Beijing account for approximately 31% of the general population (23). NASH is an important intermediate link between simple fatty liver development and cirrhosis and even malignant tumors (24). However, there are still no approved pharmaceutical therapies (25), and the underlying mechanisms for NASH are not fully understood.

CHCHD2 was first identified and submitted to the NCBI GenBank database as hepatitis C virus non-structural protein 2 trans-regulated protein by our research group in 2004 (https://www.ncbi.nlm.nih.gov/nuccore/AY605046). CHCHD2 gene locus mutations are closely related to Parkinson’s disease and other neurodegenerative diseases (26, 27). It participates in various cancers, including renal cell carcinoma, non–small cell lung cancer, and breast cancer (28–30).

CHCHD2 is located in both the nucleus and mitochondria. Most mechanistic studies have focused on its function as a mitochondrial protein, including regulating oxidative stress response, electron transport, mitochondrial morphology, cristae structure, and apoptosis (22). However, its function as a transcription factor has received much less attention. CHCHD2 was reported to bind with an oxygen-responsive element and regulate isoform 2 (predominantly lung) of cytochrome c oxidase subunit 4 (5). Moreover, CHCHD2 expression levels in the nuclei were increased by 4% oxygen, which indicated its function as a hypoxia response gene (5). We performed ChIP sequencing to systematically characterize its role as a transcription factor. As shown in Figure 1C, more than 90% of these peaks were localized in the promoter regions. Consistent with a previous study that demonstrated that CHCHD2 can regulate its own expression (5), we found that CHCHD2 binds to its own promoter (Supplemental Table 1). ChIP sequencing implied that CHCHD2 may regulate endoplasmic reticulum function, mitochondrial function, autophagy, and proteolysis as a transcription factor. More importantly, potential target genes of CHCHD2 are involved in NAFLD and hepatocellular carcinoma. We and others have found that increased CHCHD2 expression is closely related to hepatocellular carcinoma (6, 31). However, it is unclear whether CHCHD2 participates in the occurrence and development of NAFLD. In this study, we found that the expression level of CHCHD2 was greatly increased in patients with NAFLD and was positively associated with fibrosis. In addition, CHCHD2 expression levels were increased in NASH mouse models. These data indicate that the effects of CHCHD2 on NASH may be mediated by its function as a transcription factor.

We developed CHCHD2-knockout mice to study its function and found that CHCHD2 deletion attenuated fibrosis in NASH livers. In addition, TAA-induced liver fibrosis was greatly improved by absence of CHCHD2. In contrast, CHCHD2 overexpression led to increased liver fibrosis in mice fed a normal or MCD/HFD diet. This indicates that CHCHD2 is a potential target for liver fibrosis. CHCHD2 binds to promoters of Notch pathway genes and activates Notch signaling, as evidenced by increased *Hes1* and *Heyl* expression. The interaction of hepatocytes with HSCs is important for fibrotic generation in NASH livers (8). The activation of Notch signaling in hepatocytes activates HSCs by increasing OPN production, which indicates that the Notch/OPN pathway in hepatocytes mediates liver fibrosis in NASH (8, 10). In addition to Notch activation, we found that OPN was upregulated by CHCHD2 overexpression. We also found Notch inhibition attenuated CHCHD2 overexpression–induced liver fibrosis in vivo and in vitro. Therefore, increased CHCHD2 levels in hepatocytes promoted liver fibrosis by Notch/OPN signaling in NASH livers. Although our data indicate that CHCHD2 may regulate Notch signaling as a transcription factor, the underlying mechanisms need to be studied in depth in our future study. Moreover, Notch signaling plays important roles in multiple cell types and organs, including gastrointestinal tract (32). The in vitro system we used demonstrated the effects of Notch signaling on CHCHD2-induced OPN expression in hepatocytes and subsequent HSC activation. The effect of DAPT in vivo was not limited to hepatocytes by systemic delivery, which is a limitation of our study.

We next investigated the regulatory mechanisms of CHCHD2 expression in NASH liver. We found that lipotoxicity and inflammation increased CHCHD2 expression in hepatocytes. The Hippo pathway and its core transcription factors are involved in development, tissue homeostasis, cancers, cardiovascular diseases, and metabolism (11, 22). They also play important roles in the development of NAFLD and liver fibrosis. High clinical NAS scores are associated with Cyr61, a TEAD target gene (13). Increased YAP/TAZ activity promotes liver fibrosis (13) and hepatobiliary carcinogenesis in NAFLD (14). The interaction of hepatocyte TAZ with TEADs may upregulate Indian hedgehog signaling molecule expression to promote NASH fibrosis (15). Both lipotoxicity and inflammation increase YAP/TAZ-TEAD activity in hepatocytes (6, 21). In our study, we found that both the mRNA and protein expression of *Tead1* were greatly increased in livers of mice with NASH, with subtle changes in *Tead2* and *4*, and decreased *Tead3* levels. These data highlight that in addition to increased YAP/TAZ activity, increased TEAD1 expression is also responsible for the increased TEAD activity in NASH.

We also found that pharmacological inhibition of the interaction between YAP and TEADs by verteporfin or knockdown of *Tead1* by siRNA substantially decreased CHCHD2 expression in hepatocytes. Thus, our data indicate that elevated CHCHD2 expression is related to increased TEAD1 in NASH livers. A recent study found that myofibroblast YAP/TAZ is dispensable for liver fibrosis in mice and suggests that other cell types are targeted by verteporfin during liver fibrogenesis (33). Consistent with this finding, our data indicate that verteporfin targets hepatocytes to reduce CHCHD2 expression and improve liver fibrosis. YAP/TAZ-Notch signaling crosstalk exists in the liver (34); according to our data, CHCHD2 may act as a hub to link the Hippo pathway with Notch signaling in hepatocytes. However, the mRNA level of *Chchd2* in the liver was not changed by MCD/HFD feeding, and in hepatocytes, its mRNA level was also not affected by palmitate or LPS treatment. In addition, verteporfin treatment inhibited CHCHD2 expression only at the protein level and not at the mRNA level. These data indicate that CHCHD2 was not directly regulated by TEADs at the transcription level, and other YAP/TAZ-TEAD target genes may be involved in this process, which requires further exploration.

In our study, we focused on CHCHD2 expression in hepatocytes. From the immunohistochemical staining of CHCHD2 in human samples, we can see that except for hepatocytes, CHCHD2 in some nonparenchymal cells was also upregulated in NASH. It remains to be seen whether CHCHD2 in other types of liver cells (such as HSCs, macrophages, endothelial cells) plays a role in NASH, and this should be a focus of future work. In addition, the role of mitochondrial CHCHD2 in NASH also needs to be investigated in the future.

In conclusion, we found that lipotoxicity and inflammation lead to CHCHD2 elevation in NASH livers by increasing YAP/TAZ-TEAD activity. Increased CHCHD2 promotes liver fibrosis in NASH mice, which may be mediated by activation of the Notch/OPN pathway in hepatocytes.

## Methods

### Liver specimens from patients with NAFLD.

We collected liver sample sections from Beijing Ditan Hospital, Capital Medical University. Liver samples were obtained from 38 patients with biopsy-proven NAFLD/NASH and 4 patients with normal liver. Serological indicators of these 4 patients suggested suspected liver disease, whereas they were proved to have normal livers after biopsy. All patients were diagnosed by a clinician, and the diagnosis was confirmed by histopathology. The liver tissue sections were analyzed independently by 2 hepatopathologists who were blinded to demographic and clinical data according to the NASH-CRN system (steatosis was scored from 0 to 3, lobular inflammation was scored from 0 to 3, ballooning was scored from 0 to 2, and liver fibrosis was scored from 0 to 4). Patients with any form of viral hepatitis, drug-induced liver disease, alcoholic liver disease (drinking history of >5 years, daily intake of ethanol of ≥40 g for male patients and ≥20 g for female patients, or intake of >80 g of ethanol per day for 2 weeks), autoimmune liver disease, cholestatic liver disease, or hereditary metabolic liver disease were excluded.

### Animals.

CHCHD2-knockout mice were generated using the CRISPR/Cas9 system. Guide RNAs flanking exon 1 and exon 3 of *Chchd2* were microinjected with Cas9 mRNA into fertilized eggs of C57BL/6 background mice at the Institute of Zoology, Chinese Academy of Sciences. In the study, 10-week-old male CHCHD2-knockout mice and littermate control mice were fed with an MCD/HFD for 4 weeks to induce NASH (35, 36) or ND (both from Medicience). CHCHD2-knockout mice and littermate controls were intraperitoneally injected with TAA (T8700, Solarbio) or saline (control group) for 7 weeks. The mice were injected with TAA 3 times a week for the 7-week duration, starting with 50 mg/kg (dose 1 and 2), 100 mg/kg (dose 3 and 4), 200 mg/kg (dose 5 through 9), 300 mg/kg (dose 10 through 15), and 400 mg/kg (dose 16 through 21) (37).

For the FPC diet–induced NASH model, 8-week-old wild-type male mice (C57BL/6; from Beijing Vital River Laboratory Animal Technology Co., Ltd.) were fed the FPC diet or ND for 24 weeks (15, 17) (Medicience).

For overexpression of CHCHD2 specifically in hepatocytes, 8-week-old wild-type male mice (C57BL/6) were injected with the AAV-CHCHD2-flag bearing *TBG* promoter, and control mice were injected with control AAV (1 *×* 10^11^ viral genomes/mouse; GeneChem Co., Ltd.). Then, 10 days after AAV injection, mice were fed with an MCD/HFD or ND diet for 4 weeks.

For Notch inhibition in vivo, mice were injected with AAV-CHCHD2 (bearing *TBG* promoter), and control mice were injected with control AAV. Ten days after AAV injection, mice were fed with an MCD/HFD diet for 4 weeks. During the NASH diet feeding period, mice were intraperitoneally injected with a Notch inhibitor DAPT (10 mg/kg; HY-13027, MedChemExpress) or the control solvent 3 times a week (38).

After treatments, the mice were sacrificed, and the plasma and liver were collected for analysis. All mice were housed in a temperature-controlled environment with 12-hour light/12-hour dark cycle and received food and water ad libitum.

### Primary murine hepatocyte culture and treatment.

Primary murine hepatocytes were isolated using collagenase I (MilliporeSigma), as described previously (39). Briefly, 5- to 6-week-old male C57BL/6 mice were anesthetized. Then, the liver was perfused with heparin, solution I (Krebs solution + 0.1 mM EGTA), and solution II (Krebs solution + 2.74 mM CaCl_2_ + 0.05% collagenase I). The perfused liver was passed through a filter (38 μm diameter) (Shangyu five star stamping screen factory) by flushing with RPMI 1640 medium. Hepatocytes were collected and cultured in RPMI 1640.

For CHCHD2 overexpression, primary murine hepatocytes were treated with 10 multiplicity of infection of Ad-CHCHD2 or Ad-Ctrl for 48 hours. For Notch inhibition, hepatocytes were pretreated with 5 μM DAPT or 5 μM IMR-1 (HY-100431, MedChemExpress) for 24 hours and then treated with Ad-CHCHD2 or Ad-Ctrl for another 48 hours. For palmitate or LPS treatment, the cells were treated with 100 μM palmitate or 100 ng/mL LPS for 48 hours with or without pretreatment with 1 nM verteporfin (sc-475698, Santa Cruz Biotechnology) for 0.5 hours. For TEAD1 knockdown, cells were treated with si-Tead1 (sense: 5′-GCUCGCCAAUGUGUGAAUAUATT-3′; antisense: 5′-UAUAUUCACACAUUGGCGAGCTT-3′) for 48 hours. To study the stability of CHCHD2 protein, hepatocytes were treated with 50 ng/mL CHX (HY-12320, MedChemExpress) for 0, 1, 3, 6, or 9 hours.

### ChIP sequencing.

Human CHCHD2 was overexpressed in primary mouse hepatocytes by adenovirus. ChIP analysis was performed using an anti-CHCHD2 antibody (catalog HPA027407, MilliporeSigma), and sequencing was performed by LC-BIO Bio-tech Ltd. Briefly, the DNA libraries were amplified for 15 cycles and sequenced using an Illumina NovaSeq 6000 with a single-end 2 × 150 sequencing mode. High-quality, clean reads were obtained from raw reads by removing sequencing adapters, short reads (length < 35 bp), and low-quality reads using Cutadapt (v1.9.1) and Trimmomatic (v0.35) (40), and the quality was controlled by FastQC (41). The clean reads were mapped to the mouse genome (assembly GRCm38). Peak annotation of gene features was performed using the ChIPseeker R package (42). The ChIP-sequencing data have been deposited in NCBI Sequence Read Archive database (BioProject number PRJNA889227).

Please refer to the Supplemental Methods for detailed materials and methods.

### Statistics.

All data are presented as mean ± SEM unless otherwise indicated and analyzed by unpaired 2-tailed *t* test or 1-way ANOVA if more than 2 groups were compared. Statistical significance was set at *P* < 0.05. The effects of LPS and CHX on CHCHD2 protein level were analyzed using 2-way ANOVA. Pearson’s correlation was performed to analyze the association between CHCHD2 and TEAD1 expression in the livers of patients.

### Study approval.

All protocols and animal studies were performed in accordance with the *Guide for the Care and Use of Laboratory Animals* by the US NIH (NIH Publication No. 85-23; National Academies Press, 2011) and approved by the Institutional Animal Care and Use Committee of Tianjin Medical University (Tianjin, China). The human study conforms to the ethical guidelines of the 1975 Declaration of Helsinki as reflected in a priori approval by the Ethics Committee of Beijing Ditan Hospital (No. 2021-047-01). No donor organs were obtained from executed prisoners or other institutionalized persons.

## Author contributions

YL and W Xiu contributed to the concept and design, data acquisition, analysis and interpretation of data, and drafting of the article. JX, XC, GW, JD, LS, BL, W Xie, and GP contributed to data acquisition of the article. QW and CW contributed to the concept and design, data acquisition, analysis and interpretation of data, and drafting of the article. QW and CW are the guarantors of the work. All authors approved the final version. The authorship order of co–first authors was assigned according to alphabetical order of their last names.

## Supplementary Material

Supplemental data

## Figures and Tables

**Table 1:**
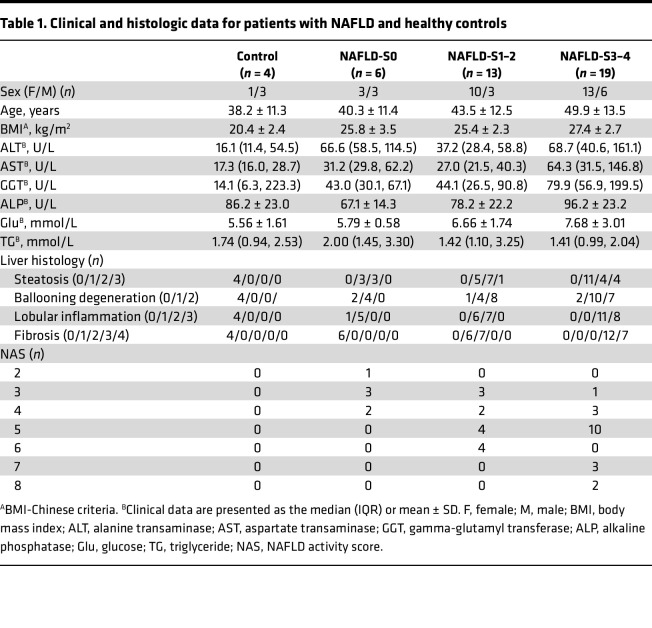
Clinical and histologic data for patients with NAFLD and healthy controls

## Version 1 12/08/2022

Electronic publication

## Version 2 12/09/2022

Corrections to Table 1 and Figure 3C
