# Supplementary material for: Increased CHCHD2 expression promotes liver fibrosis in nonalcoholic steatohepatitis via Notch/osteopontin signaling
Source: JCI Insight. 2022 Dec 8;7(23):e162402. doi: 10.1172/jci.insight.162402 (PMC9746920; doi:10.1172/jci.insight.162402)

Supplemental data

Materials and Methods

LX-2 cell culture and treatment

LX-2 (human hepatic stellate cell line) cells (from Procell Life Science&Technology Co.,Ltd., Wuhan) were cultured in Dulbecco's Modified Eagle's medium (DMEM) containing 10% fetal bovine serum. To study the crosstalk between CHCHD2-overexpressed hepatocyte and HSCs, the primary hepatocytes were transfected with si-Spp1 (sense: 5'-GAGGUCAAAGUCUAGGAGUUUTT-3'; antisense: 5'-AAACUCCUAGACUUUGACCUCTT-3') or negative control (NC) for 6 h and then were treated with CHCHD2-expressing adenovirus (Ad-CHCHD2) or control adenovirus (Ad-Ctrl) for another 42 h. Three hours after the adenovirus were added into the medium, the medium was changed into adenovirus-free medium. Then the conditioned medium was collected. The LX-2 cells were treated with the indicated conditioned medium for 24 h after serum starved for 24 h. LX-2 cells were also treated with conditioned medium from CHCHD2-overexpressed hepatocytes with or without pretreatment of Notch inhibitor DAPT or IMR-1.

Western blotting

Liver tissue or hepatocytes were lysed by incubating with cell lysis buffer (R0010, Solarbio, Beijing) containing an EDTA-free protease inhibitor cocktail (Roche, Basel, Kanton Basel) for 30 min on ice. Nuclear and mitochondrial proteins were isolated using a nuclear extraction kit and mitochondrial extraction kit, respectively (both from

Applygen Technologies, Beijing).

The protein concentrations of the samples were quantified using a BCA protein assay kit (Thermo Fisher, Waltham, MA), and equal amounts of protein from each sample were loaded onto gels and separated by sodium dodecyl sulfate-polyacrylamide gel electrophoresis (SDS-PAGE). After separation was complete, the proteins in the gel were transferred onto polyvinylidene fluoride (PVDF) membranes, and the membrane was blocked with 5% Difco Skim Milk for 1 h at room temperature. Membranes were subsequently incubated with anti-CHCHD2 (HPA027407, Sigma, Darmstadt, Hesse), anti-eIF5 (sc-28309, Santa Cruz, Dallas, TX), anti-COL3A1 (22734-1-AP, Proteintech, Wuhan), anti-Flag (14793, Cell Signaling Technology, Danvers, MA,), anti-OPN (22952-1-AP, Proteintech, Wuhan), anti-TEAD1 (610923, BD Biosciences, Franklin Lake, NJ), anti-Lamin B1 (66095, Proteintech, Wuhan), anti-COX Ⅳ (4844S, Cell Signaling Technology, Danvers, MA,), anti-Caspase3 (19677, Proteintech, Wuhan), or anti-β-actin (66009-1-Ig, Proteintech, Wuhan) antibodies at 4 °C overnight and then with the appropriate secondary antibody at room temperature for 1 h. The immunoblots were detected by enhanced chemiluminescence (Sigma Aldrich, Darmstadt, Hesse).

qPCR assay

Total RNA from mouse livers and primary hepatocytes was extracted using RNA extraction kits (Transgene, Beijing). Then, the RNA was reverse transcribed to cDNA for qPCR (Thermo Scientific, Waltham, MA). Sequences of the primers used for

qPCR are listed in Supplementary Table 2.

Oil red O staining

After fixing with 4% paraformaldehyde, liver samples were embedded in Optimal Cutting Temperature compound for frozen sectioning. Liver sections were stained with oil red O staining solution for 30 min, and nuclei were stained with hematoxylin.

H&E, Masson's trichome and Sirius red staining

Liver tissues were fixed with 4% paraformaldehyde and embedded in paraffin wax, and sections of 5 μm were stained with H&E, Masson's trichome, or Sirius red staining using a standard protocol, and then analyzed by light microscopy.

Immunohistochemistry and immunofluorescence staining

Immunohistochemical staining of F4/80, CHCHD2 and TEAD1 in human and/or mouse liver sections was performed using anti-F4/80 (70076, Cell Signaling Technology, Danvers, MA), anti-CHCHD2 (HPA027407, Sigma, Darmstadt, Hesse) or anti-TEAD1 (13283-1-AP, Proteintech, Wuhan) antibodies, respectively, on a Leica Bond RX with the Bond Polymer Refine Detection Kit (DS9800; Leica). The integrated optical density of CHCHD2 or nucleus TEAD1 in human liver sections was calculated by using Image-Pro Plus 6.0.

Immunofluorescence staining of CHCHD2 in mouse liver sections was performed using anti-CHCHD2 and anti-HNF4a (ab41898, Abcam, Cambridge) antibodies.

Determination of plasma ALT/AST levels

ALT/AST levels in the plasma were analyzed at the Beijing Ditan Hospital, Capital Medical University.

Determination of hepatic triglycerides and cholesterol content

Hepatic triglycerides and cholesterol were extracted as previously described (1). They were then determined using a triglyceride and cholesterol determination kits, respectively (BioSino Bio-Technology & Science, Beijing).

Reference

1. Wang C, Liu W, Yao L, Zhang X, Ye C, Jiang H, et al. Hydroxyeicosapentaenoic acids and epoxyeicosatetraenoic acids attenuate early occurrence of nonalcoholic fatty liver disease. Br J Pharmacol 2017;174:2358-2372.

Supplemental Figure Legends

Supplemental Figure 1. CHCHD2 was upregulated in NASH mouse liver.

(A) Wild-type mice were fed a normal diet (ND) or FPC-diet for 24 weeks: western blot analysis of protein level of CHCHD2 in liver. n = 4–5 per group. **(B-C)** Wild-type mice were fed a normal diet (ND) or a methionine/choline-deficient plus high-fat diet (MCD/HFD) for 4 weeks: (B) analysis of protein levels of COX Ⅳ and Lamin B1 in isolated nuclear and mitochondrial protein; n = 3 per group; (C) The immunofluorescence staining of CHCHD2 and HNF4α; n = 4 per group. *$p < 0.05$. *p* value was from unpaired *t-test*.

Supplemental Figure 2. Body, tissue weight and liver morphology of CHCHD2 knockout mice.

CHCHD2 knockout mice and the littermate control were fed with a ND diet: **(A)** western blot analysis of protein level of CHCHD2 in liver (n = 3 per group); **(B)** H&E staining of liver sections (n = 3 per group); **(C)** body weight of the mice (n=11-10 per group); **(D)** liver, iWAT, and eWAT weight of the mice (n = 3 per group). * $p < 0.05$. *p* values were from unpaired *t-test*.

Supplemental Figure 3. The effects of CHCHD2 knockout on NASH mouse livers

CHCHD2 knockout mice and the littermate control were fed with an MCD/HFD for 4 weeks (related to Figure 3): **(A)** body and liver weight of the mice; **(B)** western blot analysis of protein level of cleaved caspase 3 in liver; **(C)** TUNEL staining of apoptotic cells in liver (n = 8-7 per group). *p* values were from unpaired *t-test*.

Supplemental Figure 4. The expression level of fibrotic markers in TAA-treated mouse livers (related to Fig. 3).

CHCHD2 knockout mice and the littermate control were treated with TAA for 7 weeks: qPCR analysis of the mRNA levels of *Des*, *Col1a1*, *Col1a3*, *Timp1* and *Tgfb* in liver (n = 6-7 per group). *$p < 0.05$. *p* values were from unpaired *t-test*.

Supplemental Figure 5. The expression of exogenous CHCHD2 and body and liver weight of CHCHD2 overexpressed mice.

(A) Mice were injected with AAV-CHCHD2-flag with TBG promoter or control AAV. The exogenous CHCHD2 in liver, eWAT, iWAT and pancreas were evidenced by flag expression by western blotting. **(B)** The body and liver weight of CHCHD2 hepatic-specific overexpressed and control mice fed with a ND or MCD/HFD diet (n = 12-13 per group). *p* values were from unpaired *t-test*.

Supplemental Figure 6. The expression of Notch pathway genes in CHCHD2-overexpressing hepatocytes.

CHCHD2 was overexpressed in primary mouse hepatocytes by adenovirus: qPCR analysis of *Notch2*, *Notch4*, *Psen1*, *Psenen*, *Crebbp*, *Rbpj*, *dvl1* and *Ctbp1*. (n = 5 independent experiments). * $p < 0.05$. *p* values were from unpaired *t-test*.

Supplemental Figure 7. The knockdown of *Spp1* in hepatocyte by siRNA

The primary murine hepatocytes were transfected with 50 pM of *Spp1* siRNA (si-Spp1) or negative control (NC) for 48h: qPCR analysis of mRNA level of *Spp1* (n=3 independent experiments）. * $p < 0.05$. *p* value was from unpaired *t-test*.

Supplemental Figure 8. The mRNA levels of *Chchd2* in palmitate or LPS treated hepatocyte; the mRNA levels of *Tead2-4* in NASH mouse liver and TEAD1 expression level in human liver samples.

(A) The primary murine hepatocytes were treated with 100 μM palmitate for 48 h or 100 ng/mL LPS for 48 h: qPCR analysis of mRNA level of *Chchd2* (n = 5 independent experiments). (**B**) The primary murine hepatocytes were treated with 0.5 or 1 nM verteporfin for 48 h: qPCR analysis of mRNA level of *Chchd2* (n = 5 independent experiments). **(C)** qPCR analysis of mRNA levels of *Tead2-4* in livers of mice fed with ND or MCD/HFD diet (n = 12-13 per group). **(D)** Representative immunohistochemical staining of TEAD1 of liver sections of normal control (NC) and NAFLD patients (related to Figure 8I). * $p < 0.05$. (A) and (C) p values were from unpaired *t-test*; (B) p value was from a one-way ANOVA with a post hoc Fisher's least significant difference (LSD) test.

Supplementary Table 1. The information of ChIP peaks annotated as CHCHD2 itself or Notch pathway genes

**gene Id**	**transcript Id**	**symbol**	**distance to TSS**	**annotation**	**fold change**	**-log10pvalue**	**-log10qvalue**
ENSMUSG00000070493	ENSMUST00000131645	*Chchd2*	0	Promoter (<=1kb)	3.13283	5.9758	2.58248
ENSMUSG00000015468	ENSMUST00000156724	*Notch4*	200	Promoter (<=1kb)	4.70731	9.81417	5.79208
ENSMUSG00000027878	ENSMUST00000198324	*Notch2*	0	Promoter (<=1kb)	5.91455	11.8314	7.5184
ENSMUSG00000019969	ENSMUST00000041806	*Psen1*	0	Promoter (<=1kb)	4.25899	8.26141	4.49118
ENSMUSG00000036835	ENSMUST00000207747	*Psenen*	0	Promoter (<=1kb)	3.69123	8.05139	4.32068
ENSMUSG00000053040	ENSMUST00000169282	*Aph1c*	0	Promoter (<=1kb)	3.59564	5.41361	2.12294
ENSMUSG00000022528	ENSMUST00000023171	*Hes1*	0	Promoter (<=1kb)	6.84282	17.0519	12.084
ENSMUSG00000022528	ENSMUST00000160592	*Hes1*	0	Promoter (<=1kb)	5.34296	14.4335	9.78029
ENSMUSG00000039191	ENSMUST00000113865	*Rbpj*	0	Promoter (<=1kb)	3.91603	9.15153	5.2186
ENSMUSG00000039191	ENSMUST00000037618	*Rbpj*	-842	Promoter (<=1kb)	3.00028	5.26695	2.00472
ENSMUSG00000022521	ENSMUST00000023165	*Crebbp*	-18	Promoter (<=1kb)	3.63268	6.91945	3.35308
ENSMUSG00000029071	ENSMUST00000030948	*Dvl1*	0	Promoter (<=1kb)	4.33374	10.5167	6.39866
ENSMUSG00000021224	ENSMUST00000117217	*Numb*	0	Promoter (<=1kb)	3.77581	7.491	3.85396
ENSMUSG00000037373	ENSMUST00000079746	*Ctbp1*	0	Promoter (<=1kb)	6.1544	15.5643	10.7738
ENSMUSG00000028800	ENSMUST00000102597	*Hdac1*	0	Promoter (<=1kb)	3.63268	6.91945	3.35308
ENSMUSG00000046876	ENSMUST00000167708	*Atxn1*	455	Promoter (<=1kb)	5.24985	9.86552	5.83611
ENSMUSG00000000708	ENSMUST00000000724	*Kat2b*	0	Promoter (<=1kb)	3.81068	6.79597	3.25395

Supplementary Table 2. List of oligonucleotide primer pairs used in qPCR.

Target Gene	Sense Primer	Antisense Primer
Chchd2(m)	5'-GCAGGATTGCAAATGGTTTAATG-3'	5'-GATCTCTTTCACGGCCATTCC-3'
Col3a1(m)	5'-CTGTAACATGGAAACTGGGGAAA-3'	5'-CCATAGCTGAACTGAAAACCACC-3'
Des(m)	5'-CTAAAGGATGAGATGGCCCG-3'	5'-GAAGGTCTGGATAGGAAGGTTG -3'
Col1a1(m)	5'- ATGTGCCACTCTGACTGGAA-3'	5'- TCCATCGGTCATGCTCTCTC-3'
Timp1(m)	5'-CCCTTTGCATCTCTGGCATC-3'	5'-GCATTTCCCACAGCCTTGAA-3'
Cnn2(m)	5'-GGAAGACACATTTGGCCCAG-3'	5'-TAGGTGTCCGGATGCACTTT-3'
Tead1(m)	5'-GAGCGACTCGGCAGATAAGC-3'	5'-CCACACGGCGGATAGATAGC-3'
Tead2(m)	5'-GAAGACGAGAACGCGAAAGC-3'	5'-GATGAGCTGTGCCGAAGACA-3'
Tead3(m)	5’-GCAAGATGTACGGTCGAAATGA-3’	5’-TCTTCCGAGCTAGAACCTGTATG-3’
Tead4(m)	5'-ACAATGATGCAGAGGGTGTATG-3'	5'-TCCTCCGTCAGGATAATTTTGC-3'
Tgfb(m)	5'-'ACCGCAACAACGCCATCTA-3	5'- GCCCTGTATTCCGTCTCCTT-3'
Hes1(m)	5'-CAACACGACACCGGACAAAC-3'	5'-CGGAGGTGCTTCACAGTCAT-3'
18S(m/h)	5'-GGAAGGGCACCACCAGGAGT-3'	5'-TGCAGCCCCGGACATCTAAG-3'
Actb(m)	5'-GCTCTGGCTCCTAGCACCAT-3'	5'-GGGCCGGACTCATCGTACT-3'
Rbpj(m)	5'-GGCTACATCCATTACGGGCA-3'	5'-AGAACGTGTACTCGGCCTTG-3'
Crebbp(m)	5'-TGGAAGAACTGCACACGACA-3'	5'-AAGTGGCATTCTGTTGCCCT-3'
Psenen(m)	5'-ATGAACTTGGAGCGGGTATCC-3'	5'-CGAGGAACGCCTCTCTGAAG-3'
Psen1(m)	5'-GAATGACAGCCAAGAACGGC-3'	5'-CACGGGGACAAAGAGCATGA-3'
Ctbp1(m)	5'-CTGGGGATCTAGGCATCGC-3'	5'-GTTCGTCGGTACAGGTTCAGG-3'
Dvl1(m)	5'-GCTATGGTACGAGTCCCTGC-3'	5'-CACTCTTCACAGTCAGCGGT-3'
Notch4(m)	5'-CCAGGCTATGAGGGACAGAA-3'	5'-GTAGAAGGCGTTGGCTAAAGAG-3'
Notch2(m)	5'-ATTGGGTTGATGATGAAGGA-3'	5'-CTGAGGAGGAGTGAGTGCC-3'
Heyl(m)	5'-AACCCGGCGGAATTTGTTG-3'	5'-GGATTGGGACTATGCTCCTGG-3'
COL1A1(h)	5'-GAGGGCCAAGACGAAGACATC-3'	5'-CAGATCACGTCATCGCACAAC-3'
TGFB(h)	5'-CTTTCCTGCTTCTCATGGCC-3'	5'-TCCAGGCTCCAAATGTAGGG-3'
TIMP1(h)	5'-AGAGTGTCTGCGGATACTTCC-3'	5'-CCAACAGTGTAGGTCTTGGTG-3'
CCN2(h)	5'-GTGGAGTATGTACCGACGGC-3'	5'-GCAGGCACAGGTCTTGATGA-3'

m: Mus musculus; h: Homo sapiens.

Supplemental Figure 1

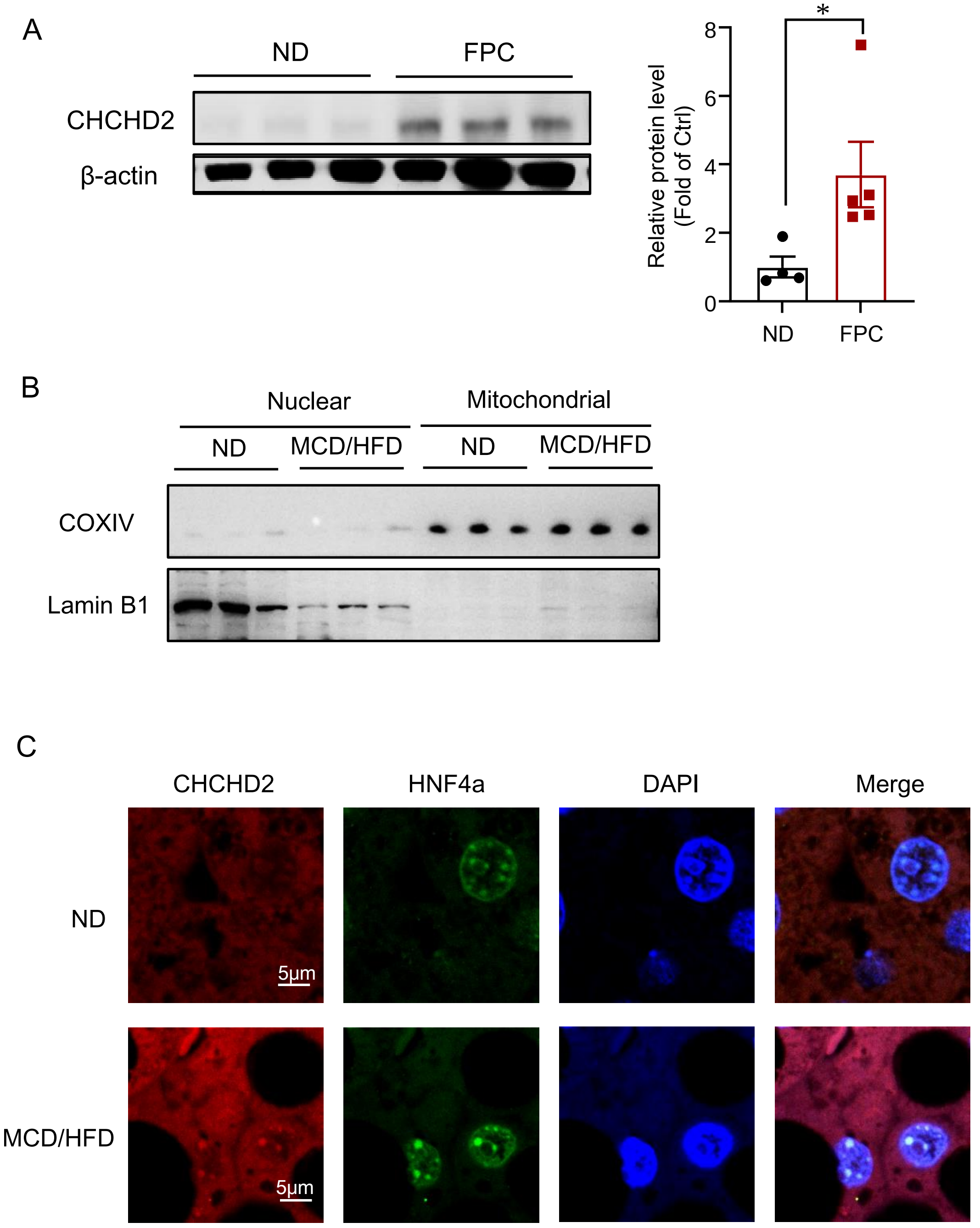

Supplemental Figure 2

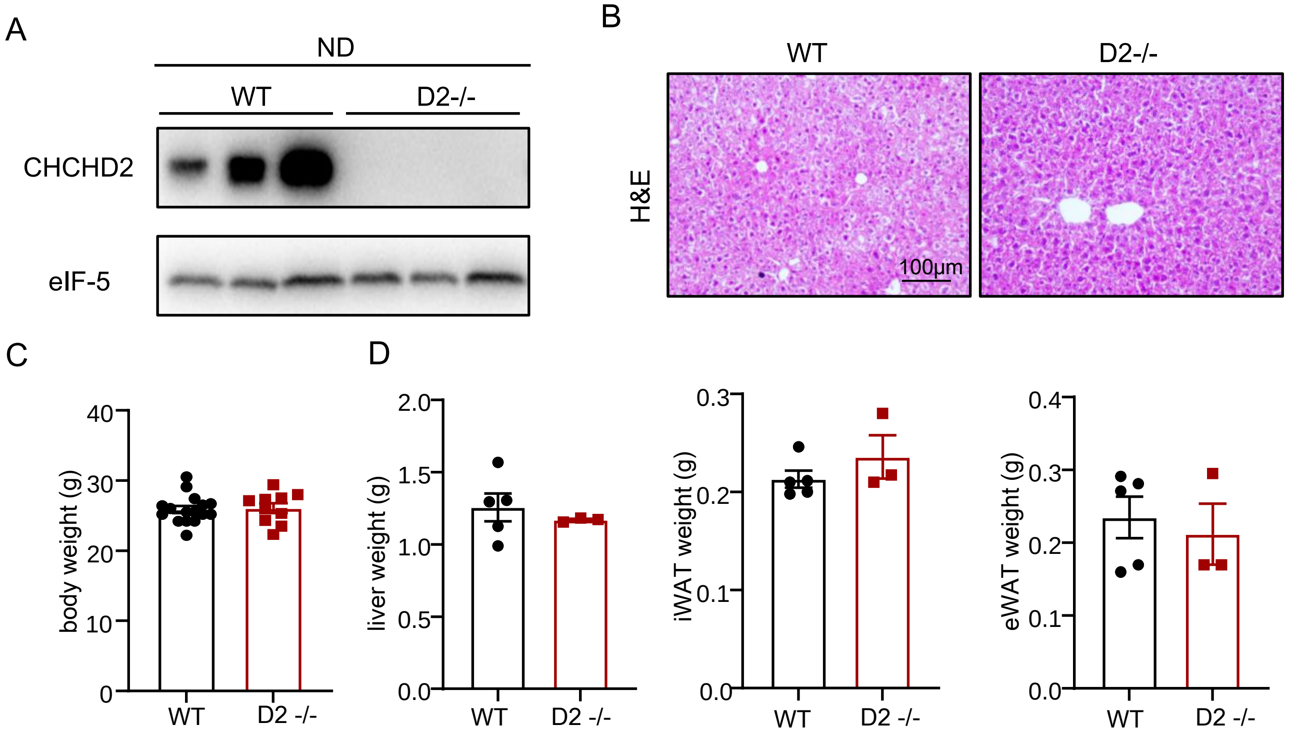

Supplemental Figure 3

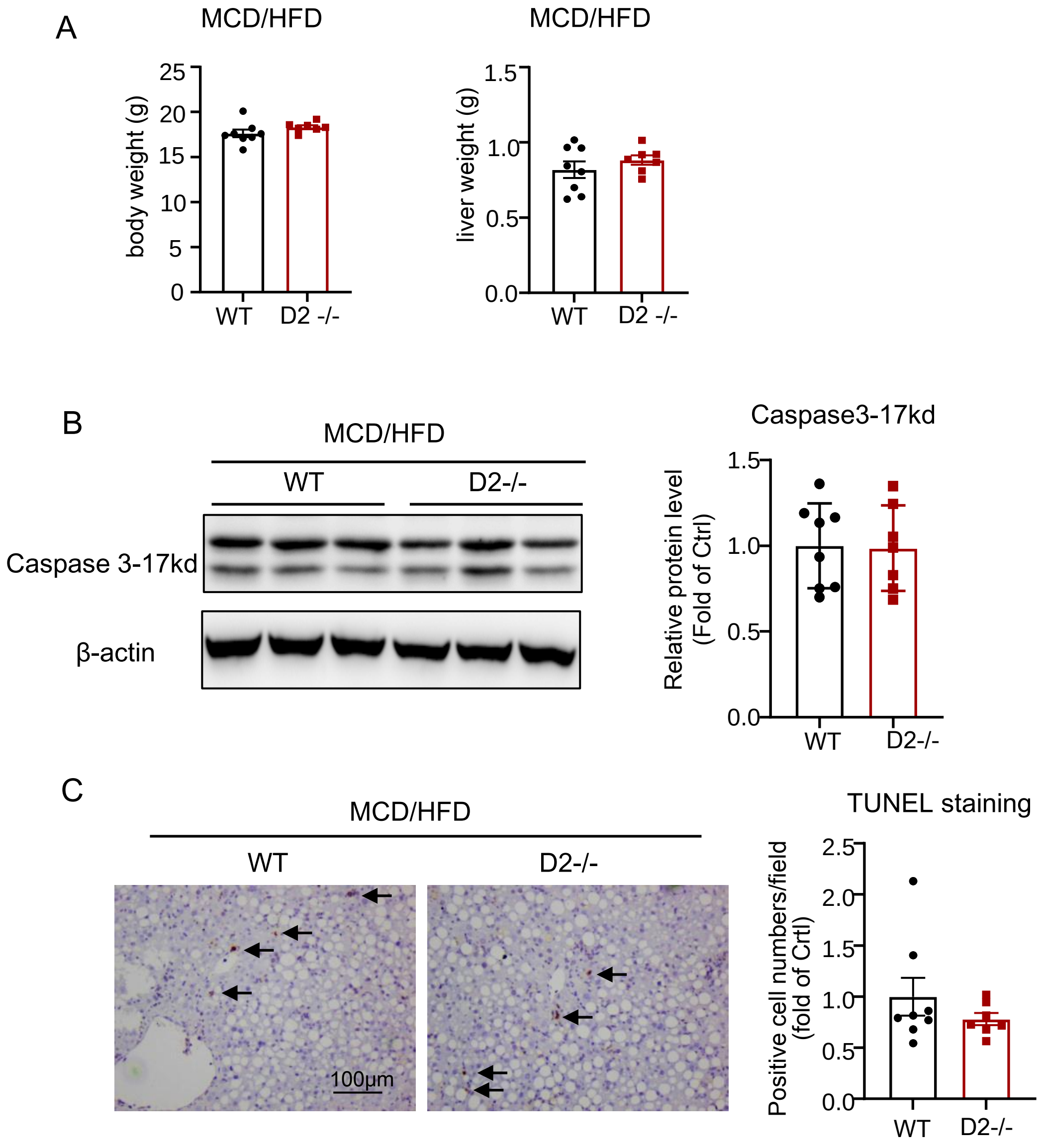

Supplemental Figure 4

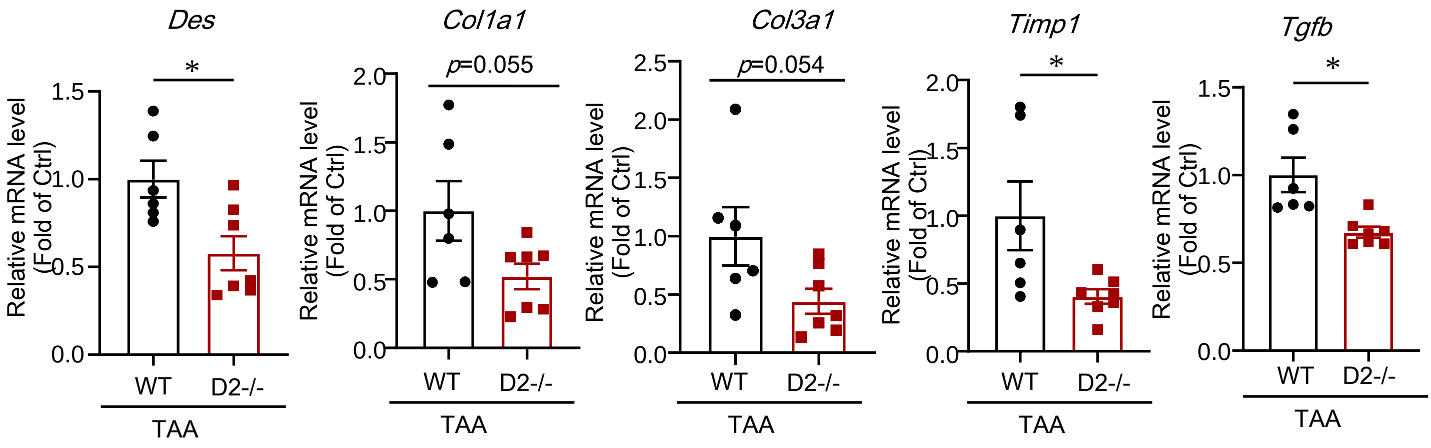

Supplemental Figure 5

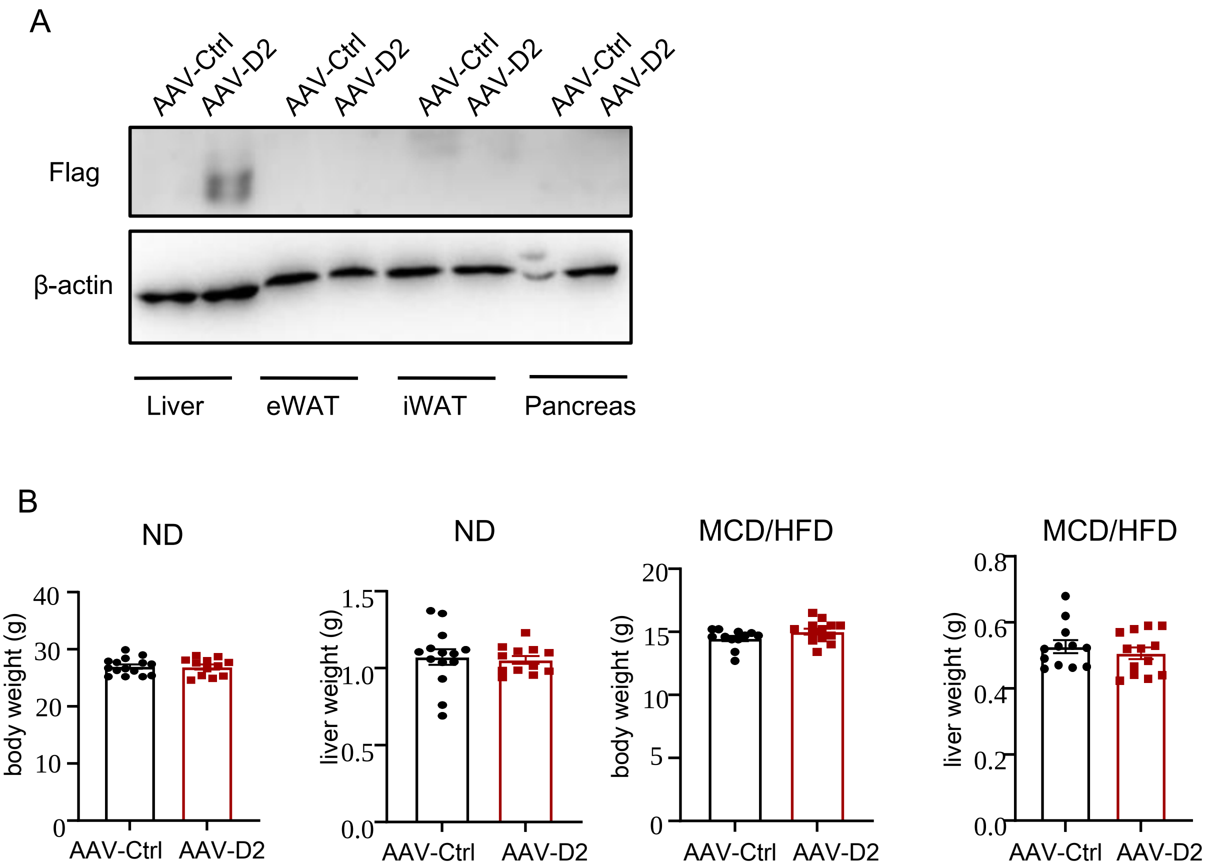

Supplemental Figure 6

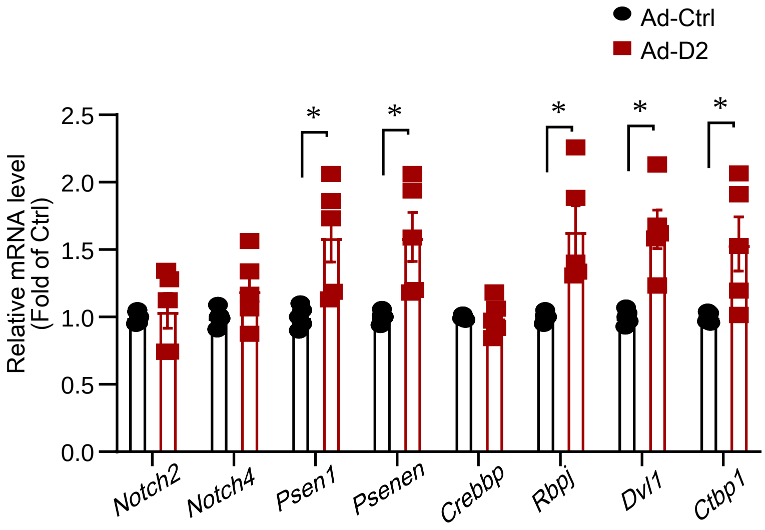

Supplemental Figure 7

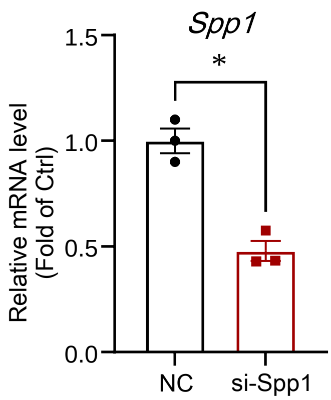

Supplemental Figure 8

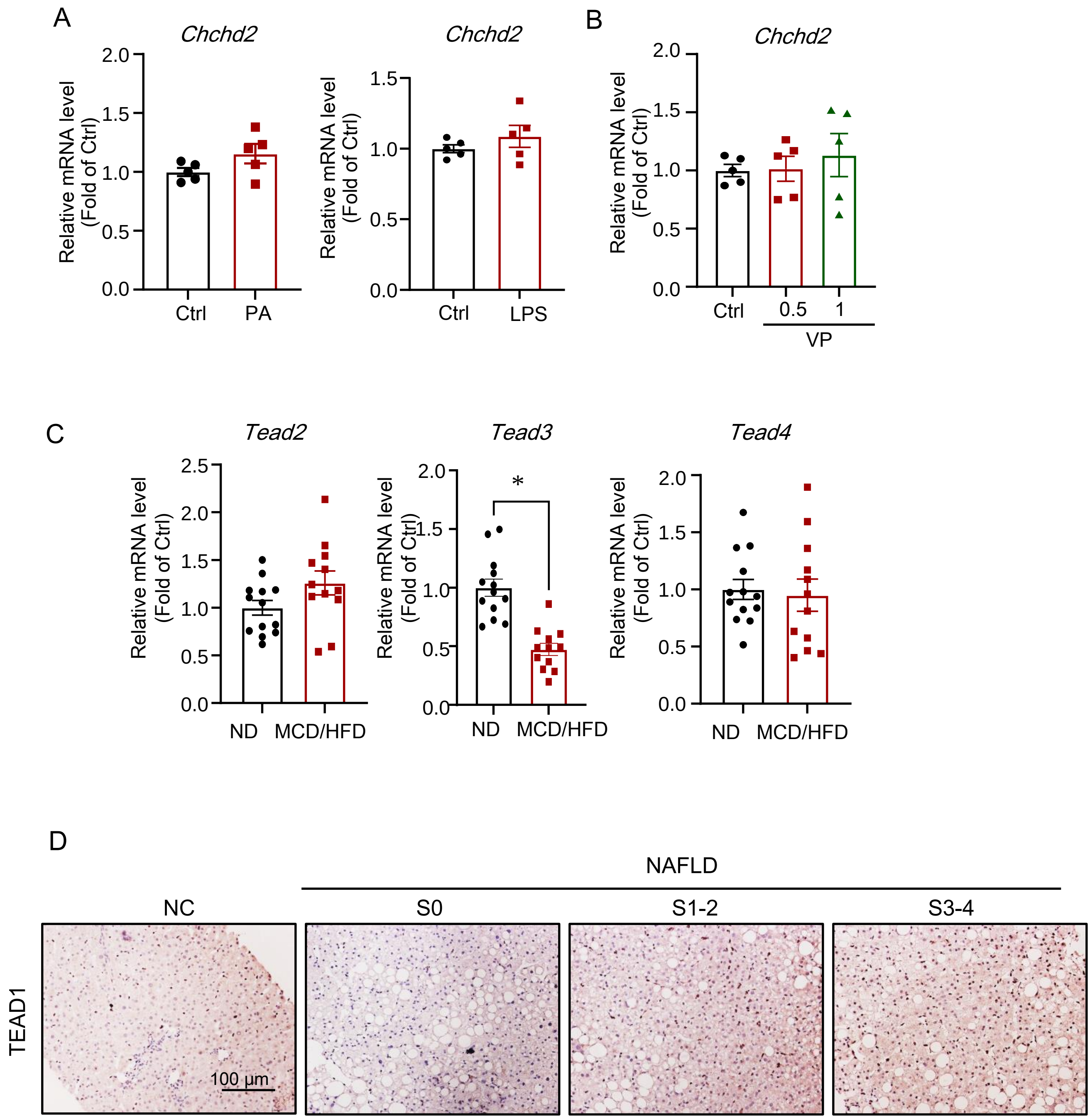

Supplement: Supplemental data [file jciinsight-7-162402-s259.pdf]
